# Genetic landscape and PD-L1 expression in Epstein–Barr virus-associated gastric cancer according to the histological pattern

**DOI:** 10.1038/s41598-023-45930-6

**Published:** 2023-11-09

**Authors:** Ji Hyun Park, Hee Jin Cho, Jeonghwa Seo, Ki Bum Park, Yong Hwan Kwon, Han Ik Bae, An Na Seo, Moonsik Kim

**Affiliations:** 1 Department of Pathology, Yonsei University College of Medicine, Seoul, Republic of Korea; 2 Department of Biomedical Convergence Science and Technology, Kyungpook National University, Daegu, Republic of Korea; 3 Cell and Matrix Research Institute, Kyungpook National University, Daegu, Republic of Korea; 4 Department of Statistics, Kyungpook National University Chilgok Hospital, Kyungpook National University, Daegu, Republic of Korea; 5 Department of Surgery, School of Medicine, Kyungpook National University Chilgok Hospital, Kyungpook National University, Daegu, Republic of Korea; 6 Department of Internal Medicine, School of Medicine, Kyungpook National University Chilgok Hospital, Kyungpook National University, Daegu, Republic of Korea; 7 Department of Pathology, School of Medicine, Kyungpook National University Chilgok Hospital, Kyungpook National University, Daegu, 41405 Republic of Korea

**Keywords:** Cancer, Genetics

## Abstract

Epstein–Barr virus (EBV)-associated gastric cancer (EBVaGC) is a distinct molecular subtype of gastric cancer. This study aims to investigate genomic and clinicopathological characteristics of EBVaGC according to the histological pattern. We retrospectively collected 18 specimens of surgically resected EBVaGCs. Whole-exome sequencing was performed for all cases. Moreover, PD-L1 expression and tumor-infiltrating lymphocyte (TIL) percentage were investigated. Among 18 EBVaGCs, 10 cases were of intestinal histology, 3 were of poorly cohesive histology, and the remaining 5 were of gastric carcinoma with lymphoid stroma histology. Whole-exome sequencing revealed that EBVaGCs with intestinal histology harbored pathogenic mutations known to frequently occur in tubular or papillary adenocarcinoma, including *TP53, KRAS*, *FBXW7*, *MUC6*, *ERBB2*, *CTNNB1*, and *ERBB2* amplifications. One patient with poorly cohesive carcinoma histology harbored a *CDH1* mutation. Patients with EBVaGCs with intestinal or poorly cohesive carcinoma histology frequently harbored driver mutations other than *PIK3CA*, whereas those with EBVaGCs with gastric carcinoma with lymphoid stroma histology lacked other driver mutations. Moreover, the histological pattern of EBVaGCs was significantly associated with the levels of TILs (*P* = 0.005) and combined positive score (*P* = 0.027). In conclusion, patients with EBVaGCs with different histological patterns exhibited distinct genetic alteration, PD-L1 expression, and degree of TILs.

## Introduction

Globally, stomach cancer has been recognized as one of the most common malignancies. In South Korea, the incidence of gastric cancer is the fourth highest (10.8%) among cancer types, followed by thyroid (11.8%), lung (11.7%) and colon (11.2%) cancers^1^. Epstein–Barr virus (EBV)—a member of the herpes virus family—is one of the most common pathological viruses in humans^2^. Although *Helicobacter pylori* infection is a major cause of gastric cancer^3^, a subtype of gastric cancer is associated with EBV infection and presents with distinct clinicopathological characteristics^4^. EBV-associated gastric cancer (EBVaGC) comprises 2%–20% of gastric cancer cases depending on the region, with a global average of 8.9%^5–7^. EBVaGC is preferentially located in the upper-to-middle third of the stomach^8^. EBVaGC is usually associated with a better prognosis than other subtypes of gastric cancer^9^. Histologically, it usually demonstrates irregular cords, nests, and sheets of glands embedded in a dense lymphocytic infiltration, and it is called gastric carcinoma with lymphoid stroma (GCLS)^10^. However, EBVaGCs histologically resembling conventional intestinal or poorly cohesive carcinoma (PCC) have also been reported in the literature^11^.

Although the Cancer Genome Atlas group (TCGA) performed comprehensive genomic profiling of gastric cancer, only a few cases (*n* = 26) were used for the genetic analysis of EBVaGC compared with those for the analysis of other molecular subtypes of gastric cancer^12^. Thus, the genomic characterization of EBVaGC needs further investigation. Meanwhile, immune checkpoint inhibitors have been widely used to treat patients with cancer. Recently, nivolumab—an anti-programmed death protein 1 (PD-1) antibody—has received FDA approval for the first-line treatment of advanced stage gastric cancer^13^. Moreover, patients with EBVaGCs, which are usually associated with a lymphocyte-rich tumor microenvironment, have been recognized as good candidates for immunotherapy^14^. However, the response of such patients to immunotherapy can be affected by diverse histological patterns and tumor microenvironment of these cancers^15^.

In this study, we comprehensively investigated the genetic characteristics of EBVaGCs and Programmed death-ligand 1 (PD-L1) expression according to their histological pattern in an East Asian cohort.

## Results

### Clinicopathological characteristics of the patient cohort

**Figure 1:**
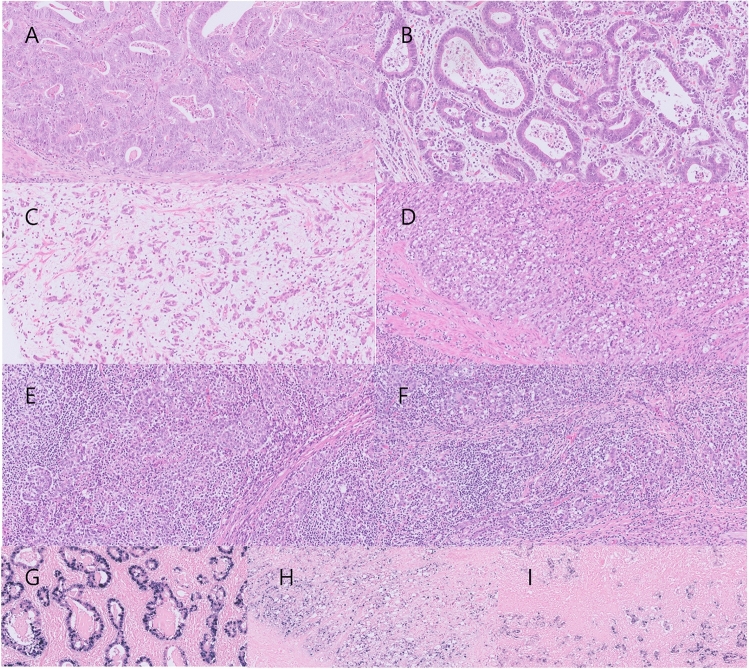
Representative images of EBVaGCs according to their histological pattern. Hematoxylin and eosin images (**A**, **B**) of intestinal histology, (**C**, **D**) PCC histology, and (**E**, **F**) GCLS histology. (**G**) EBER in situ hybridization result of (**B**). (**H**) EBER in situ hybridization result of (**D**). (**I**) EBER in situ hybridization result of (**F**) Original magnifications: (**A**–**I**): × 200.

Table 1 shows the clinicopathological characteristics of the 18 EBVaGC cases evaluated in this study, according to the dominant histological pattern. The mean age of the patients was 64.9 years. There were 15 men (72.2%) and three women (27.8%). Ten cases (55.6%) had intestinal histology, five cases (27.8%) had GCLS histology, and the remaining three cases (16.6%) had PCC histology (Fig. 1). Interestingly, only one case with GCLS histology was found among advanced gastric cancers (pT2N0; Stage 1B). The histological patterns of EBVaGCs were significantly associated with perineural invasion (*P* = 0.019) and tumor stage (*P* = 0.023). Detailed information of the patient cohort is shown in Table S1.

**Table 1:** Clinicopathological characteristics of EBVaGC according to the histological pattern.

Variables		All (*n* = 18)	Intestinal (*n* = 10)	GCLS (*n* = 5)	PCC (*n* = 3)	*P*-value
Age, years (mean ± SD)		64.9 ± 5.4	64.0 ± 5.7	67.2 ± 2.4	64.3 ± 8.5	0.575
Sex	Male	15 (83.3%)	9 (90.0%)	4 (80.0%)	2 (66.7%)	0.619
	Female	3 (16.7%)	1 (10.0%)	1 (20.0%)	1 (33.3%)	
LVI	Present	8 (44.4%)	6 (60.0%)	0 (0.0%)	2 (66.7%)	0.061
	Absent	10 (55.6%)	4 (40.0%)	5 (100.0%)	1 (33.3%)	
PNI	Present	7 (38.9%)	4 (40.0%)	0 (0.0%)	3 (100.0%)	0.019
	Absent	11 (61.1%)	6 (60.0%)	5 (100.0%)	0 (0.0%)	
LN metastasis	Present	8 (44.4%)	6 (60.0%)	0 (0.0%)	2 (66.7%)	0.061
	Absent	10 (55.6%)	4 (40.0%)	5 (100.0%)	1 (33.3%)	
Stage (AJCC 8th)	I	7 (38.9%)	2 (20.0%)	5 (100.0%)	0 (0.0%)	0.023
	II	4 (22.2%)	3 (30.0%)	0 (0.0%)	1 (33.3%)	
	III	7 (38.9%)	5 (50.0%)	0 (0.0%)	2 (66.7%)	

GCLS, gastric carcinoma with lymphoid stroma; LVI, lymphovascular invasion; PCC, poorly cohesive carcinoma; PNI, perineural invasion; LN, lymph node; SD, standard deviation.

### Overall genomic characteristics of the patient cohort

Sequencing occurred at an average depth of 228 × . Overall, 30,964 somatic SNVs and INDEL mutations were identified. The median number of mutations per case was 365 (range, 226–18,219). The median tumor mutational burden (TMB) was 4.14 (range, 2.53–49.22) muts/Mb. Two cases were TMB-High. According to the microsatellite instability (MSI) polymerase chain reaction (PCR) test, all cases were microsatellite stable. Details of the somatic analysis are shown in Table S2.

### Genomic characteristics of EBVaGCs according to their histological pattern

**Figure 2:**
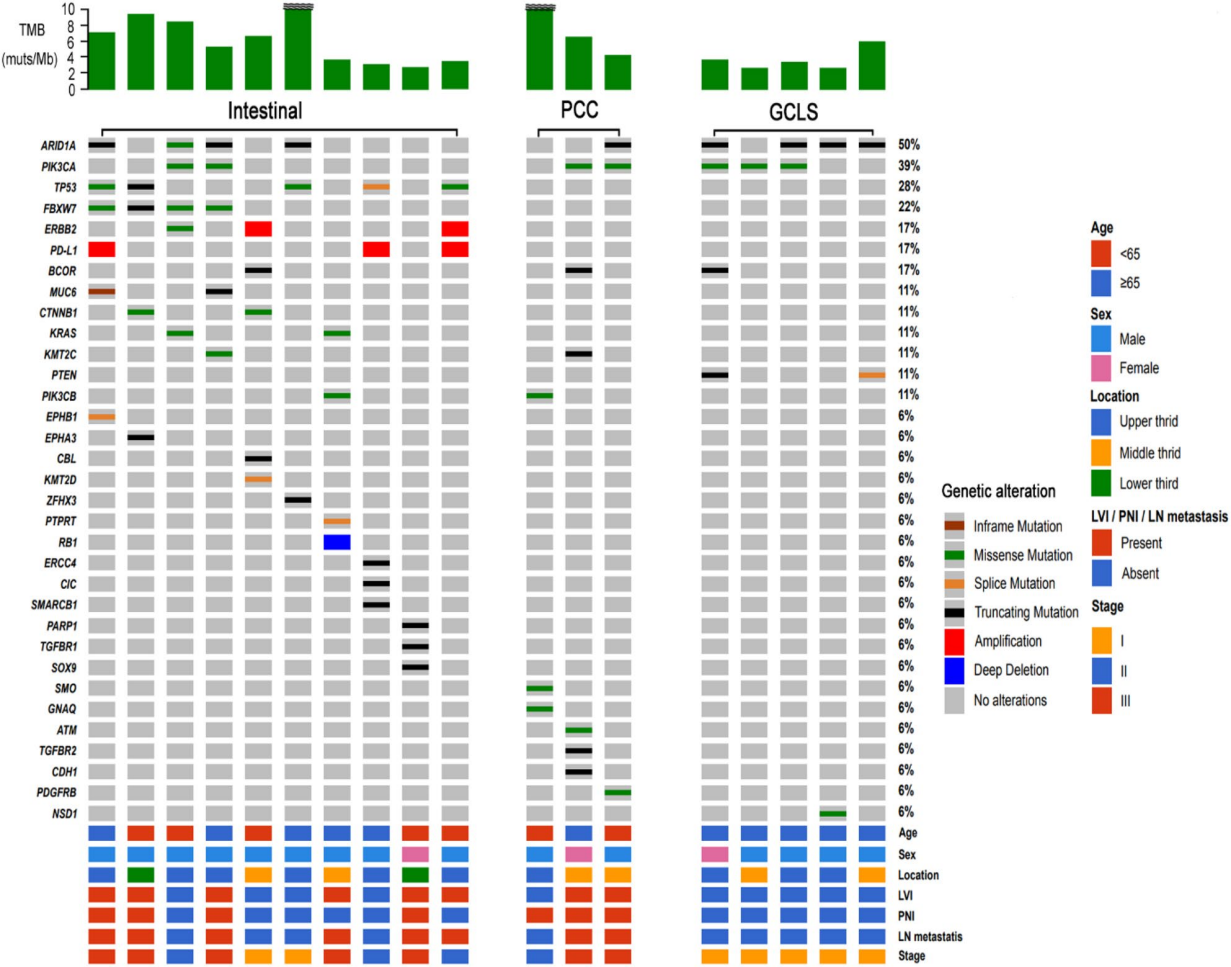
Oncoprint of EBVaGCs according to their histological pattern. TMB value of > 10 was marked by a tilde.

Figure 2 is an Oncoprinter that characterizes pathogenic alterations found in EBVaGCs. EBVaGCs with intestinal or PCC histology frequently had driver mutations other than *PIK3CA*, whereas EBVaGCs with GCLS histology lacked other driver mutations. *PIK3CA* mutations were found in seven of the 18 cases (38.9%), and *ARID1A* mutations were found in nine of the 18 cases (50%). *PD-L1* amplification was found in three cases (16.7%). *TP53* mutations were found in 5 of the 18 cases (27.8%), with higher frequencies than previously reported for EBVaGCs^12^. Furthermore, four of the five cases of *TP53*-mutant EBVaGC were advanced gastric cancer; and one case of *TP53*-mutant early gastric cancer had lymph node metastasis. EBVaGCs with intestinal histology were frequently accompanied by genetic alterations, which were known to frequently occur in conventional intestinal-type adenocarcinoma, including *KRAS*, *FBXW7*, *MUC6*, *ERBB2*, *CTNNB1*, and *ERBB2* amplification mutations^16,17^. EBVaGCs with GCLS histology were not accompanied by genetic alterations that were known to frequently occur in either intestinal-type adenocarcinoma or PCC. One case of EBVaGC with PCC histology had *a CDH1* mutation. Detailed information on the pathogenic mutations found in this study is shown in Table S3. No statistically significant mutual exclusivity or co-occurrence pattern was observed between pathogenic mutations (Table S4).

### Copy number variation (CNV) analysis in EBVaGCs

Genome-wide copy number alterations in EBVaGCs are shown in Fig. S1. *PD-L1* amplifications were found in three cases. All cases showing *PD-L1* amplification had intestinal histology. Copy number alterations were more frequently found in advanced EBVaGCs than in early EBVaGCs (Fig. S2).

### Mutational signature of EBVaGCs

**Figure 3:**
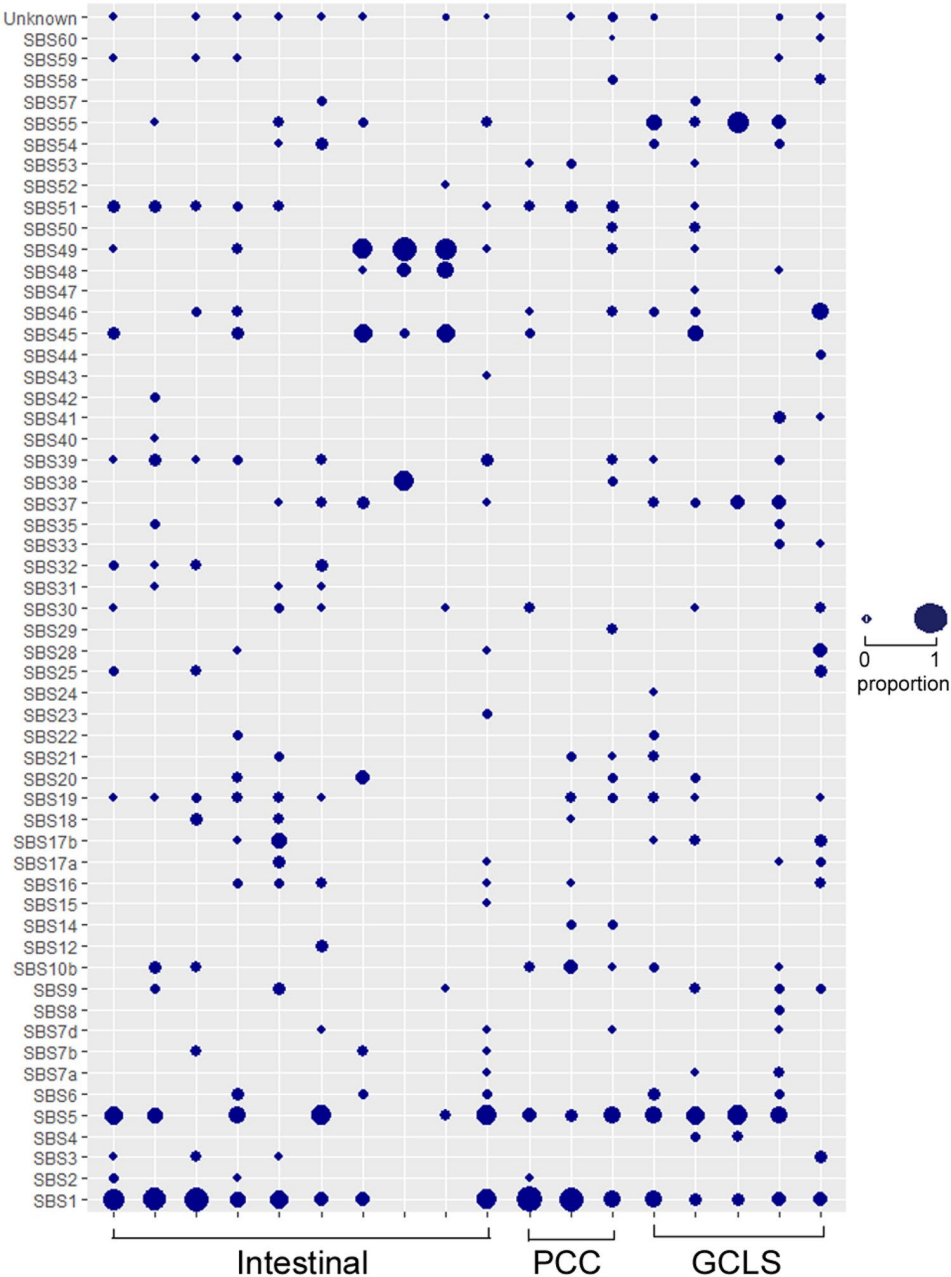
Mutational signature of EBVaGCs according to their histological pattern. The size of the circle is proportional to the portion of mutational signatures in each case.

In the mutational signature analysis, the most frequently occurring signatures were single base substitution (SBS) 1 and SBS5, which are also known as clock-like signatures (aging signatures). Other frequently occurring signatures were SBS19, SBS39, SBS30, SBS37, SBS10b, SBS16, and SBS9 (Fig. 3). No significant differences in mutational signatures were observed according to the histological pattern.

### Pathway analysis

We mapped the pathogenic mutations and CNVs to signaling pathways (Table 2). PIK3 pathway was less frequently involved in EBVaGCs with intestinal histology (*P* = 0.047). EBVaGCs with intestinal or PCC histology frequently involved pathways other than the PIK3 pathway. Further, genes involved in the receptor tyrosine kinase-rat sarcoma virus (RTK-RAS) pathway were predominantly found (6/7, 85.7%) in patients with intestinal histology. Genes involved in the wingless/integrated (Wnt) signaling pathway were exclusively found in patients with intestinal histology. Meanwhile, in patients with EBVaGCs with GCLS histology, only the phosphatidylinositol 4,5-bisphosphate (PI3K) pathway was found to be involved.

**Table 2:** Pathways involved in EBVaGC according to the histological pattern.

Pathway	All (n = 18)	Intestinal (n = 10)	GCLS (n = 5)	PCC (n = 3)	*P*-value
PI3K	10 (55.6)	3 (30.0)	4 (80.0)	3 (100.0)	0.047
RTK-RAS	7 (38.9)	6 (60.0)	0 (0.0)	1 (33.3)	0.114
Cell cycle	6 (33.3)	6 (60.0)	0 (0.0)	0 (0.0)	0.058
WNT	2 (11.1)	2 (20.0)	0 (0.0)	0 (0.0)	0.407
TGF-β	2 (11.1)	1 (10.0)	0 (0.0)	1 (33.3)	0.343
GPCR	1 (5.6)	0 (0.0)	0 (0.0)	1 (33.3)	0.167
HRD	1 (5.6)	0 (0.0)	0 (0.0)	1 (33.3)	0.167

GPCR, G protein-coupled receptor; HRD, homologous recombination deficiency; PI3K, phosphatidylinositol 4,5-bisphosphate; RTK-RAS, receptor tyrosine kinase-rat sarcoma virus; TGF-β, Transforming growth factor beta; WNT, wingless/integrated.

### PD-L1 expression and tumor-infiltrating lymphocytes (TILs) of EBVaGCs according to the histological pattern

**Figure 4:**
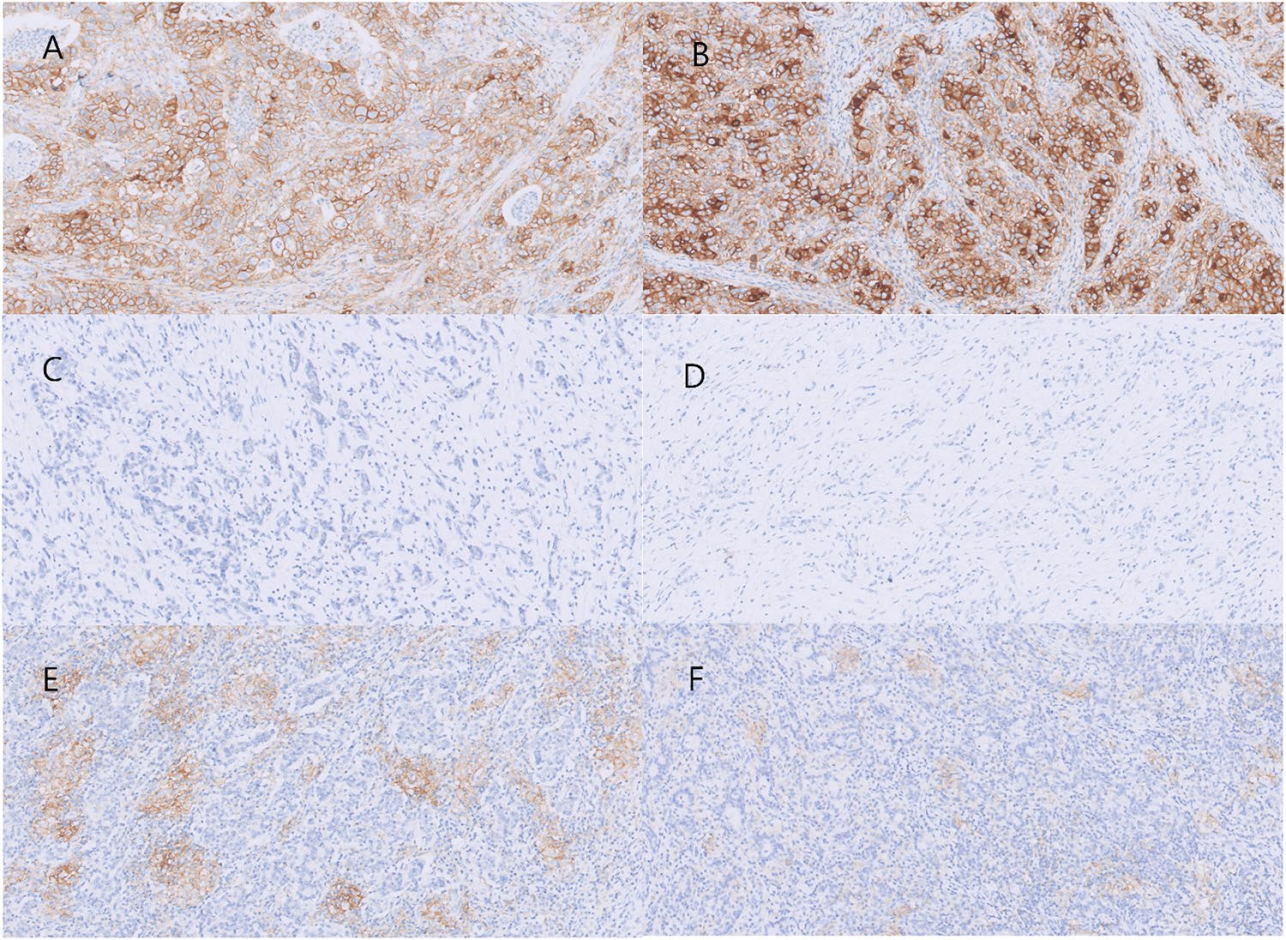
Different PD-L1 expressions of EBVaGCs according to their histological patterns. (**A**, **B**) Intestinal histology showing diffuse and strong PD-L1 expression levels on tumor cells. Both cases had PD-L1 amplification on the CNV analysis. (**C**, **D**). PCC histology showing TPS and CPS negativity. (**E**, **F**) GCLS histology. PD-L1 expression was focused on the surrounding immune cells rather than on the tumor cells. Original magnifications: (**A**–**F**): × 200.

**Figure 5:**
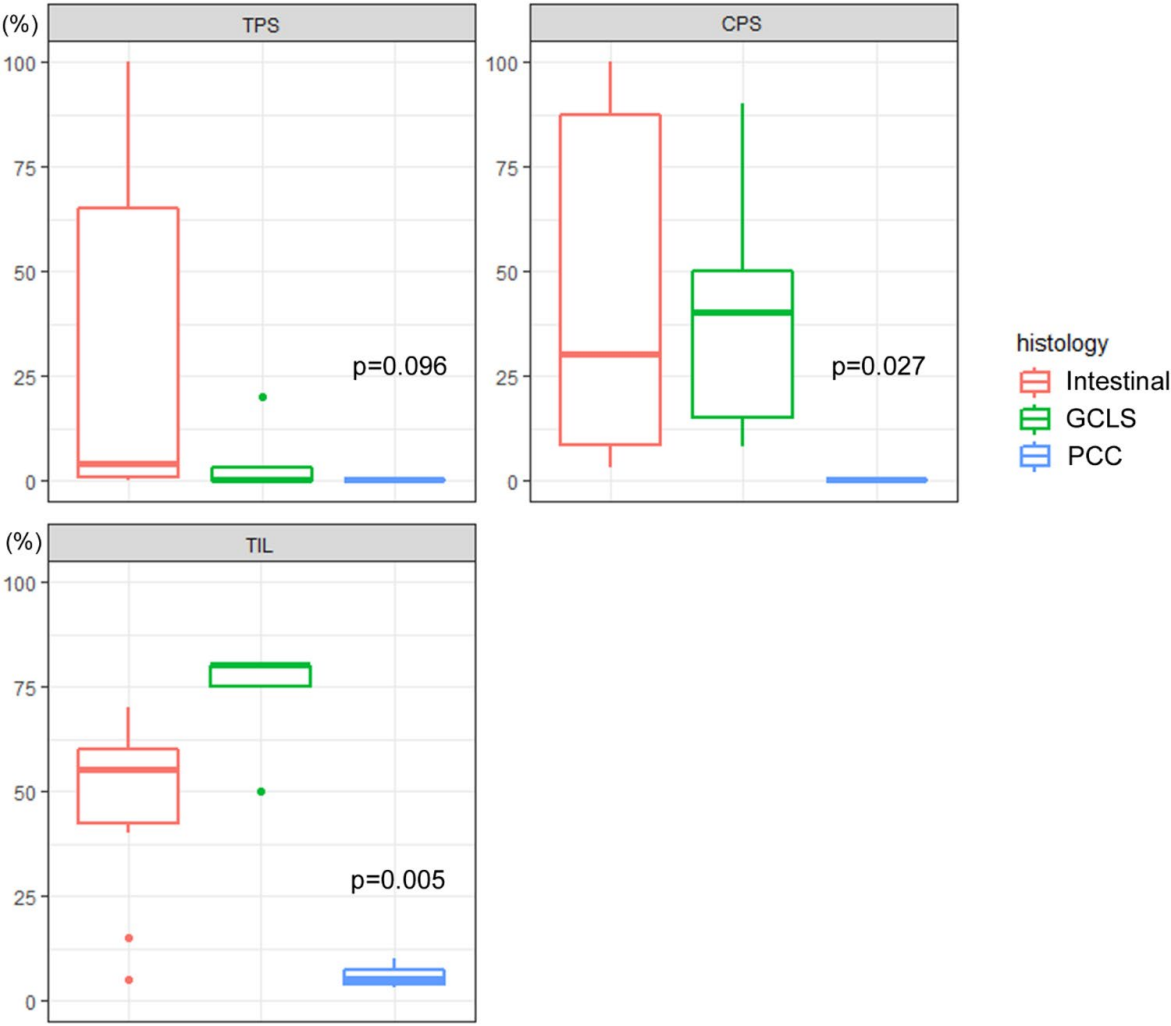
Box plot showing PD-L1 TPS, CPS, and TIL expression of EBVaGCs according to their histological pattern.

We further analyzed whether PD-L1 expression and the levels of TILs in EBVaGCs is associated with their histological pattern (Fig. 4). The combined positive score (CPS) and TIL percentage were significantly associated with the histological pattern of EBVaGCs (Fig. 5). Remarkably, all cases with PCC histology were CPS-negative. Tumor proportion score (TPS) positivity tended to be found more frequently in cases with intestinal histology (Table 3). TIL-high tumors were significantly associated with the histological pattern (*P* = 0.007) and CPS positivity (*P* = 0.019) (Table S5).

**Table 3:** TPS and CPS according to the histological pattern of EBVaGC.

Variables		All (*n* = 18)	Intestinal-type like *(n* = 10)	GCLS (*n* = 5)	PCC (*n* = 3)	*P*-value
TPS	Positive (≥ 1%)	9 (50.0%)	7 (70.0%)	2 (40.0%)	0 (0.0%)	0.091
	Negative (< 1%)	9 (50.0%)	3 (30.0%)	3 (60.0%)	3 (100.0%)	
TPS	High (≥ 50%)	4 (22.2%)	4 (40.0%)	0 (0.0%)	0 (0.0%)	0.128
	Low (< 50%)	14 (77.8%)	6 (60.0%)	5 (100.0%)	3 (100.0%)	
CPS	Positive (≥ 1)	15 (83.3%)	10 (100.0%)	5 (100.0%)	0 (0.0%)	< 0.001
	Negative (< 1)	3 (16.7%)	0 (0.0%)	0 (0.0%)	3 (100.0%)	

TPS, tumor proportion score; CPS, combined positive score.

### Comparison with TCGA cohort

We further validated the results of this study using TCGA cohort. All but one EBVaGC in TCGA cohort had advanced gastric cancer; surprisingly, among 26 EBVaGC cases in TCGA cohort, only two were diagnosed with GCLS in the original pathology report. We also independently reviewed all EBVaGC cases in TCGA cohort. On the histological review, intestinal histology were 18 cases, PCC histology were 5 cases, and GCLS histology were 2 cases (Table S6). Pathogenic mutations, including *CTNNB1*, *BRAF*, *KRAS*, *NRAS*, *TP53*, and *ERBB2* amplification, were frequently found in EBVaGCs showing intestinal histology, consistent with the findings of our study. Among the five cases with PCC histology, *RHOA* mutations were found in two cases. A significant difference in TIL percentage was also observed (*P* = 0.006) according to the histologic pattern of EBVaGCs in TCGA cohort (Fig. S3).

## Discussion

In this study, we demonstrated the distinct genetic characteristics and PD-L1 expression of EBVaGCs according to the histological pattern.

EBVaGCs with intestinal histology commonly harbored genetic alterations involving the RTK-RAS pathway and other pathways known to frequently occur in cases of tubular or papillary adenocarcinoma, which can be used as druggable targets^18–20^. Moreover, all *TP53* mutations were found in EBVaGCs with intestinal histology. Although *TP53* mutations rarely occur in EBVaGCs, some studies have reported a higher rate of *TP53* mutations in EBVaGCs^21,22^. In this study, *TP53*-mutant cases did not have *PIK3CA* mutations. He et al. suggested possible mutual exclusivity between *TP53* and *PIK3CA* mutation in EBVaGCs^22^. This may partially explain the rare *TP53* mutations in TCGA cohort (20/26 [76.9%] cases had *PIK3CA* mutations) and relatively high frequency of *TP53* mutations in our cohort (7/18 [38.9%] had *PIK3CA* mutations). The *CDH1* (this cohort) and *RHOA* (TCGA cohorts) mutations that were detected in EBVaGCs with PCC histology in TCGA cohort further support the association between genetic alterations and histological patterns^12^. Meanwhile, *ASTE1*, *FAT3*, *and* STING mutations, which have been reported to indicate clinical significance^22–24^, were not found in this study.

EBVaGCs with intestinal, PCC, and GCLS histology exhibited different patterns of PD-L1 expression and TILs. Patients with EBVaGCs with GCLS histology exhibited the highest CPS score and TIL (%), consistent with the findings of previous studies^25,26^; PD-L1 was mainly expressed on the surrounding immune cells rather than on the tumor cells. *PD-L1* amplification was found only in EBVaGCs with intestinal histology; PD-L1 expression was mainly found on tumor cells. EBVaGCs with PCC histology were all TPS- and CPS-negative, and these cases exhibited fibrous and/or myxoid stroma and significantly low amounts of TILs. Low PD-L1 expression and poor response to immune checkpoint inhibitors are a well-known phenomenon in gastric cancers with PCC histology^27^. Taken together, histological patterns might be considered to establish a treatment plan for EBVaGC.

No significant differences were found according to the histological pattern in the CNV and mutational signature analyses. However, the presence of multiple signatures detected in each case in addition to aging signatures suggests the multiplicity of mechanisms can be involved in EBVaGC evolution.

One of the limitations of this study is the relatively small number of cases analyzed. However, the clinicopathological significance of this study has been further supported by the validation based on TCGA cohort. Although EBVaGCs were classified according to the dominant histologic pattern, significant morphological and genetic intratumoral heterogeneity was reported in EBVaGC^28^. Accordingly, the majority of EBVaGCs with intestinal histology (9/10, 90.0%) and PCC histology (2/3, 66.7%) had at least focal area of the GCLS component in this cohort (Table S1). Multiregion sequencing of EBVaGCs with distinct histologic patterns might be needed to better elucidate their clonal relationship. In addition, to reduce sampling error and avoid intratumoral heterogeneity issue, biopsy samples should not be used for the subtyping of EBVaGCs. In this study, TIL levels were evaluated to assess the tumor microenvironment. Although high TIL levels are known to be associated with better response to immune checkpoint inhibitors^29^, its clinical utility is still under investigation. Performing multiplex IHC on immune cells or single-cell RNA sequencing can help better understand the complex immune microenvironment of EBVaGCs according to subtypes.

In conclusion, EBVaGCs with distinct histology were found to harbor a distinct pattern of genetic alterations. Moreover, EBVaGCs with intestinal histology frequently harbored targetable alterations. Histologically classifying EBVaGCs can help predict the response to immune checkpoint inhibitors and prognosis.

## Materials and methods

### Study population

First, we retrospectively examined 12 cases of advanced EBVaGCs, which are known to have more prognostic significance than early gastric cancer, among surgically resected and histologically confirmed 356 cases of gastric adenocarcinoma from 2019 to 2021 in the Department of Pathology, Kyungpook National University Chilgok Hospital. Further, 6 cases of early EBVaGCs among 110 consecutive cases of surgically resected gastric adenocarcinoma in 2019 were included for comparison. Of the 466 total cases of surgically resected gastric adenocarcinoma, GCLS histology was found in 37 (7.9%). Of these cases, 27 were EBVaGCs and 8 were MSI-high type. All selected cases were chemo-naive specimens. EBV-encoded RNA (EBER) in situ hybridization was routinely performed for all resected specimens of gastric cancer. The results of EBER in situ hybridization test revealed that all specimens of EBVaGCs demonstrated diffuse nuclear positivity on tumor cells. We collected clinicopathological data, including age, sex, tumor size, tumor location, lymphovascular invasion, perineural invasion, lymph node metastasis, and tumor stage from the electronic medical records of the hospital.

### Pathological evaluation

All gastric cancer specimens were fixed in 10% neutral-buffered formalin and embedded in paraffin blocks. Further, the paraffin blocks were cut into 4-μm-thick sections and stained with hematoxylin and eosin. Two independent pathologists who were experienced in gastrointestinal pathology (MSK and ANS) reviewed all available slides and a representative slide was selected for whole-exome sequencing and PD-L1 immunohistochemistry for each case. A few studies have classified the histology of EBVaGCs into GCLS, Crohn’s disease-like, and conventional adenocarcinoma-like histology largely based on the amount of lymphocytic infiltration^15,30^. However, we found that determining a clear cutoff point for the amount of lymphocytic infiltration in this three-tier system is difficult. Therefore, we classified EBVaGC histology into GCLS, intestinal-like, and PCC-like histology. GCLS histology was defined as irregular cords, nests, trabecular, or solid sheets of glands with prominent lymphocytic infiltration. Intestinal-like and PCC-like histology were defined as EBVaGCs showing either typical intestinal-like or poorly cohesive carcinoma-like histology as suggested by WHO classification^31^. Patients presenting mixed histology were categorized according to the dominant histological pattern.

### Whole-exome sequencing (WES)

A representative section of forrmalin-fixed, paraffin-embedded (FFPE) block was used for the sequencing. For each case, tumor area showing dominant histological pattern was marked and dissected for DNA extraction. A QIAamp DNA FFPE Tissue kit (Qiagen, Valencia, CA, USA) was used to extract DNA from FFPE tissue, following the manufacturer’s protocols. A NanoDrop spectrophotometer (Thermo-Fisher Scientific, Waltham, MA, USA) was used to measure the concentration and quality of DNA. WES was performed using the SureSelect V6-Post (FFPE) kit (Agilent Technologies, Santa Clara, CA, USA) and processed on a NovaSeq 6000 sequencer (Illumina, San Diego, CA, USA) to achieve a mean depth of 200 × for the tumor samples and 100 × for the matched normal samples from the lymph node tissue of the same patient. Then, sequence reads were aligned to the human reference genome hg38 using the Burrows − Wheeler Aligner-MEM algorithm^32^. Picard was used to mark duplicate or low-quality reads. The genome analysis tool kit (GATK) was used for base quality score recalibration^33^. Somatic mutation calling^34,35^, TMB analysis^36,37^, CNV analysis^38–40^, and mutational signature analysis^41,42^ were performed as previously described.

### Evaluation of TILs

As there is no consensus guideline for evaluating TILs in gastric cancer, we evaluated the percentage of TILs using breast cancer international consensus scoring recommendations^43^. One representative whole section was used for the evaluation. Briefly, only stromal TILs were counted. TILs were evaluated within the border of the tumors. We calculated the average percentage of TILs per whole section slide, not focusing on hotspots. Currently, a consensus regarding TIL-high or TIL-low does not exist in gastric cancer. Therefore, we set 50% of TILs as the cutoff point because lymphocyte-rich stroma is usually used as a term from tumors having more lymphocytes than tumor cells^15,43^.

### Evaluation of PD-L1 expression

For PD-L1 immunohistochemistry, a representative whole section of each tumor was used. PD-L1 22C3 pharmDx assay using Monoclonal Mouse Anti-PD-L1 (Clone 22C3, Agilent) was performed on each case using EnVision FLEX visualization system on Autostainer Link 48 (Agilent). During PD-L1 assessment, clinicopathological information and WES analysis results were blinded. TPS was measured using the percentage of PD-L1-expressing tumor cells among the total tumor cells. CPS was measured using the number of PD-L1-stained cells (including tumor cells, lymphocytes, and macrophages) divided by the total number of viable tumor cells and then multiplied by 100. CPS-positive was defined as CPS ≥ 1 based on KEYNOTE-059 study^44^. Although there is no consensus regarding TPS score in gastric cancer, we defined TPS-positive and TPS-high as TPS ≥ 1% and TPS ≥ 50% respectively, using TPS guideline in non-small cell lung cancer^45,46^. Discrepancies in interpretation were resolved by discussion.

### EBER in situ hybridization

For EBER in situ hybridization, an INFORM EBV-encoded RNA probe (Ventana Medical Systems, Oro Valley, AZ, USA) was used to assess the EBV status of the gastric cancer according to the manufacturer’s instructions. Each hybridization run contained positive control obtained from EBV-positive nasopharyngeal carcinoma. For all surgically resected and histologically confirmed gastric cancers, EBER in situ hybridization was performed up front to minimize the degradation of viral genetic material.

### MSI testing

PCR using five National Cancer Institute markers (i.e., BAT‐26, BAT‐25, D5S346, D17S250, and S2S123) was performed to assess the MSI status of the specimens. Representative tumor sections and matched normal tissues were used for MSI PCR testing. A DNA autosequencer (ABI 3731 Genetic Analyzer; Thermo-Fisher Scientific, Waltham, MA, USA) was used to analyze the PCR products. According to the revised Bethesda Guidelines^47^, tumors with at least two markers with unstable peaks were classified as MSI-high, tumors with one unstable marker were defined as MSI-low, and tumors with no unstable markers were designated as microsatellite stable.

### Statistical analysis

Relationships among clinicopathological parameters were evaluated using the chi-square test, Fisher’s exact test, ANOVA, Mann–Whitney U test, and Kruskal–Wallis test. *P*-values of < 0.05 were used to indicate a significant difference. Further, for the analysis of mutual exclusivity and co-occurrence of genetic mutations, Bonferroni-corrected *p*-values (*q*-value) were used. *Q*-values of < 0.1 were considered significant. All statistical analyses were performed using the R software (version 4.2).

### Ethical approval and consent to participate

The study was conducted in accordance with the guidelines of the Declaration of Helsinki and approved by the Institutional Review Board of Kyungpook National University Chilgok Hospital (No. KNUCH 2022-01-035-002). The informed written consent was also obtained from the subjects involved in this study.

### Supplementary Information

Supplementary Information.

## Data Availability

All authors confirm adherence to the policy. The data that support the findings of this study will be made available at reasonable request. Correspondence and requests for data and materials should be addressed to ANS and MSK.
