# Supplementary material for: Genetic landscape and PD-L1 expression in Epstein–Barr virus-associated gastric cancer according to the histological pattern
Source: Sci Rep. 2023 Nov 9;13:19487. doi: 10.1038/s41598-023-45930-6 (PMC10636116; doi:10.1038/s41598-023-45930-6)
Supplement: Supplementary file 1 — Supplementary Information. [file 41598_2023_45930_MOESM1_ESM.pdf]

Supplementary Materials for Genetic landscape and PD-L1 expression of Epstein–Barr Virus-Associated Gastric Cancer according to histologic pattern

Ji Hyun Park, Hee Jin Cho, Jeonghwa Seo, Ki Bum Park, Yong Hwan Kwon, Han Ik Bae, An Na Seo, and Moonsik Kim

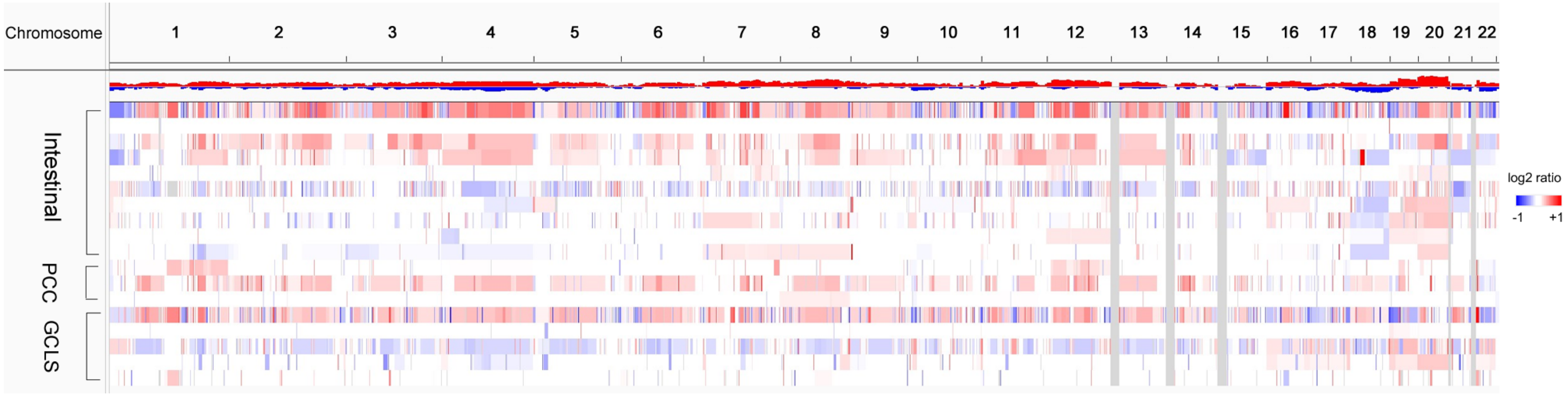

Fig. S1. Copy number analysis of EBVaGCs according to their histological pattern. The log2 ratio of tumor reads to normal reads is shown.

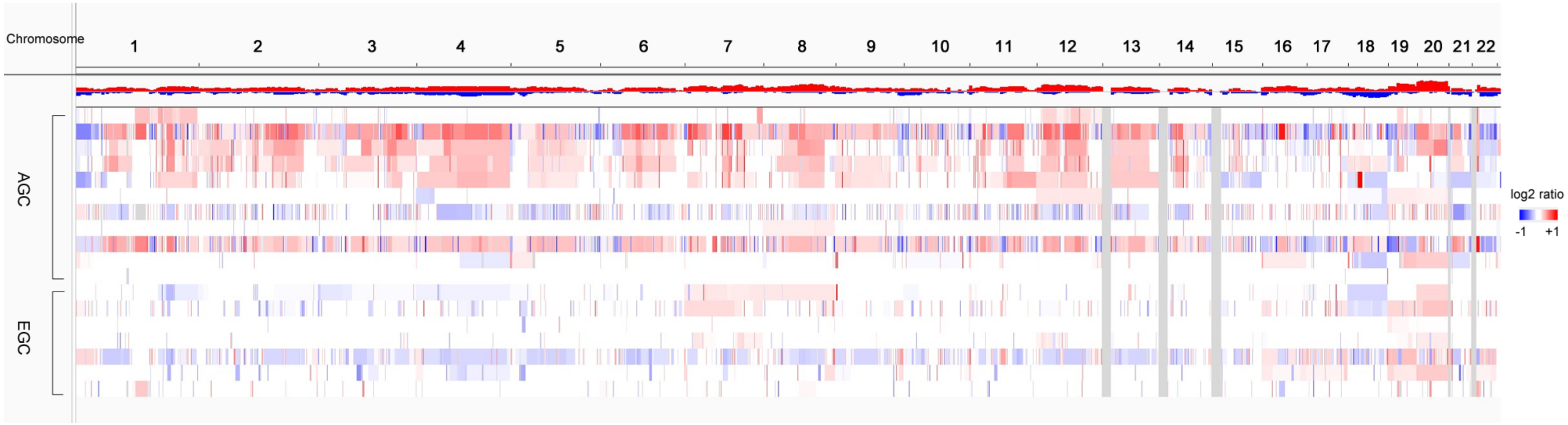

Fig. S2 Copy number analysis of EBVaGCs divided into early gastric cancer and advanced gastric cancer. Log2 ratio of tumor reads to normal reads are shown.

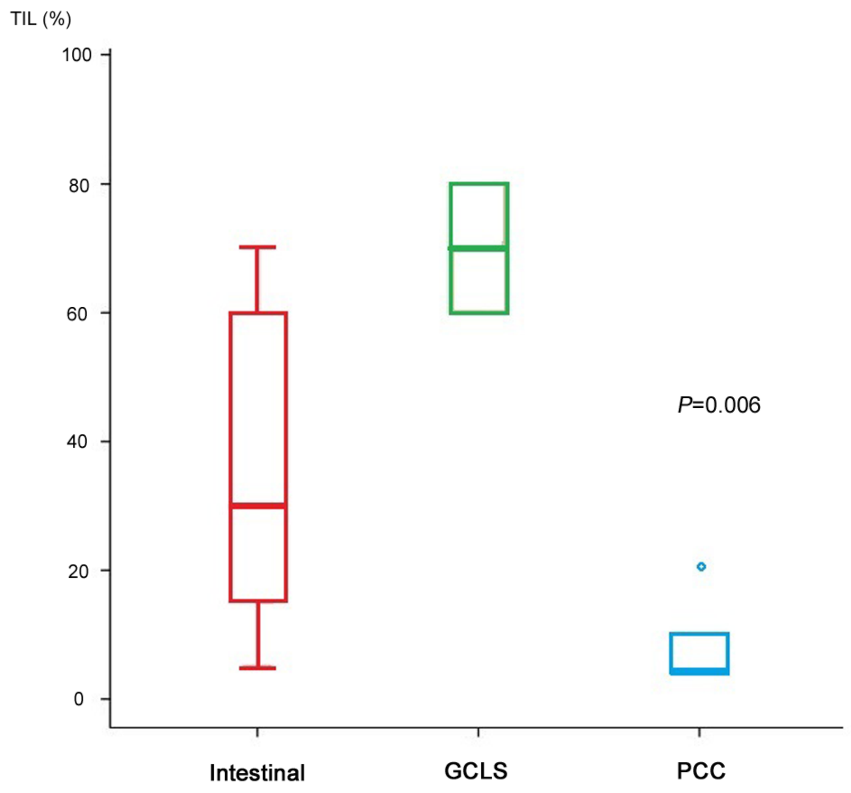

Fig. S3 Box plot showing TILs percentage of EBVaGCs according to histologic pattern (TCGA EBVaGC cohort)

Table S1. Clinicopathologic information of patients cohort

Case no.	Age, years	Sex	Mode of surgery	Tumor location	Tumor size, cm	Predominant histology	Focal GCLS component (%)	LVI	PNI	T	N	Stage
1	70	M	Total	Upper	7.5	Intestinal-type like	Y (10%)	Y	Y	4a	3b	III
2	58	M	Total	Lower	6.0	Intestinal-type like	Y (15%)	Y	Y	3	2	III
3	63	M	Subotal	Upper	4.5	Intestinal-type like	N	N	N	3	0	II
4	70	M	Total	Upper	8.5	Intestinal-type like	Y (25%)	Y	Y	3	1	III

5	55	M	Subtotal	Middle	2.2	Intestinal-type like	Y (20%)	N	N	1b	0	I
6	72	M	Total	Upper	2.8	Intestinal-type like	Y (30%)	N	N	1b	0	I
7	65	M	Subtotal	Middle	7.5	Intestinal-type like	Y (5%)	Y	N	4a	2	III
8	66	M	Total	Upper	4.2	Intestinal-type like	Y (10%)t	N	N	3	0	II
9	58	F	Total	Lower	4.0	Intestinal-type like	Y (35%)	Y	Y	3	2	III
10	63	M	Total	Upper	3.5	Intestinal-type like	Y (20%)	Y	N	1b	1	II
11	64	M	Total	Upper	3.5	PCC	Y (15%)	N	Y	3	0	II
12	73	F	Subtotal	Middle	8.7	PCC	N	Y	Y	4a	2	III

13	56	M	Total	Middle	11.1	PCC	Y (5%)	Y	Y	3	3b	III
14	71	F	Subtotal	Upper	2.2	GCLS	N/A	N	N	1b	0	I
15	68	M	Total	Middle	3.5	GCLS	N/A	N	N	2	0	I
16	66	M	Subtotal	Upper	1.8	GCLS	N/A	N	N	1b	0	I
17	65	M	Total	Upper	2.7	GCLS	N/A	N	N	1b	0	I
18	66	M	Subtotal	Middle	1.8	GCLS	N/A	N	N	1b	0	I

GCLS, gastric carcinoma with lymphoid stroma; N/A, not applicable; LVI, lymphovascular invasion; PCC, poorly cohesive carcinoma; PNI, perineural invasion

Table S2. SNV, Indel, and TMB of EBVaGCs

Case no	Number of SNV	Synonymous variant	Missense variant	Stop gained	Stop lost	Number of INDEL	Frameshift variant	Inframe deletion	TMB (muts/Mb)
1	575	101	195	11	0	59	10	4	7.06
2	675	113	272	18	1	84	13	4	9.35
3	607	108	246	9	0	80	12	2	8.45
4	434	66	121	9	0	61	12	3	5.02
5	556	86	208	8	0	51	12	2	6.83
6	18,219	3,155	2,954	53	6	1,190	51	52	49.22
7	701	99	264	8	1	74	6	2	3.97
8	4,518	954	1,746	172	1	69	5	1	3.09

9	1,197	235	437	78	0	60	5	4	2.76
10	281	43	87	4	0	39	4	3	3.37
11	757	154	292	16	0	85	11	5	10.06
12	480	75	183	7	0	56	7	1	6.59
13	365	45	135	8	0	79	10	2	4.3
14	304	41	97	11	1	35	5	1	3.65
15	260	26	73	7	1	57	4	0	2.53
16	289	20	87	8	0	45	6	1	3.35
17	226	22	68	3	0	38	5	4	2.58
18	520	56	129	7	0	78	5	3	6

Table S3. All pathogenic genomic variants found in EBVaGCs

Case no.	Chromosome	Position	Gene	Variant type	HGVS.c	Protein change	VAF
1	chr4	152,326,016	*FBXW7*	Missense mutation	c.1634A>G	p.Tyr545Cys	0.114
1	chr17	7,674,220	*TP53*	Missense mutation	c.743G>A	p.Arg248Gln	0.667
1	chr11	1,016,003	*MUC6*	Inframe deletion mutation	c.6795_6797delCTC	p.Ser2266del	0.583
1	chr1	26,761,012	*ARID1A*	Nonsense mutation	c.2077C>T	p.Arg693*	0.227
1	chr1	26,779,305	*ARID1A*	Nonsense mutation	c.5407G>T	p.Glu1803*	0.084
1	chr3	135,192,824	*EPHB1*	Splice site mutation	c.2130+1G>T		0.164
2	chr3	41,224,634	*CTNNB1*	Missense mutation	c.122C>T	p.Thr41Ile	0.172

2	chr17	7,674,230	*TP53*	Missense mutation	c.733G>A	p.Gly245Ser	0.333
2	chr3	89,341,823	*EPHA3*	Frameshift mutation	c.1041delG	p.Trp347fs	0.087
2	chr4	152,326,165	*FBXW7*	Frameshift mutation	c.1483_1484delCA	p.His495fs	0.518
2	chr17	7,676,071	*TP53*	stop_gained	c.298C>T	p.Gln100*	0.115
3	chr1	26,780,158	*ARID1A*	Missense mutation	c.6260G>A	p.Gly2087Glu	0.611
3	chr3	179,218,303	*PIK3CA*	Missense mutation	c.1633G>A	p.Glu545Lys	0.174
3	chr4	152,328,233	*FBXW7*	Missense mutation	c.1393C>T	p.Arg465Cys	0.521
3	chr12	25,245,350	*KRAS*	Missense mutation	c.35G>A	p.Gly12Asp	0.237
3	chr17	39,711,955	*ERBB2*	Missense mutation	c.929C>A	p.Ser310Tyr	0.302
4	chr3	179,199,088	*PIK3CA*	Missense mutation	c.263G>A	p.Arg88Gln	0.206
4	chr4	152,326,214	*FBXW7*	Missense mutation	c.1436G>A	p.Arg479Gln	0.105
4	chr7	152,185,587	*KMT2C*	Missense mutation	c.5053G>T	p.Ala1685Ser	0.136

4	chr1	26,731,342	*ARID1A*	Frameshift mutation	c.1542delT	p.Gln515fs	0.190
4	chr11	1,026,071	*MUC6*	Frameshift mutation	c.2616_2617insAGTA	p.Glu873fs	0.267
5	chr3	41,224,646	*CTNNB1*	Missense mutation	c.134C>T	p.Ser45Phe	0.132
5	chr11	119,285,193	*CBL*	Frameshift mutation	c.1570delT	p.Ser524fs	0.093
5	chr12	49,048,071	*KMT2D*	Splice site mutation	c.4132-2A>G		0.175
5	chrX	40,074,239	*BCOR*	Frameshift mutation	c.1103_1106delCTTC	p.Pro368fs	0.253
6	chr17	7,674,230	*TP53*	Missense mutation	c.733G>A	p.Gly245Ser	0.114
6	chr1	26,779,284	*ARID1A*	Frameshift mutation	c.5387_5391dupCAGCT	p.Ser1798fs	0.189
6	chr16	72,794,800	*ZFHX3*	stop_gained	c.7882G>T	p.Glu2628*	0.125

7	chr3	138,655,451	*PIK3CB*	Missense mutation	c.3151G>A	p.Glu1051Lys	0.165
7	chr12	25,245,286	*KRAS*	Missense mutation	c.99T>G	p.Asp33Glu	0.286
7	chr20	42,248,823	*PTPRT*	Splice site mutation	c.2234-1G>C		0.066
8	chr16	13,920,220	*ERCC4*	Nonsense mutation	c.55G>T	p.Glu19*	0.027
8	chr17	7,673,534	*TP53*	Splice site mutation	c.993+1G>T		0.474
8	chr19	42,292,464	*CIC*	Nonsense mutation	c.5900C>A	p.Ser1967*	0.434
8	chr22	23,825,325	*SMARCB1*	Nonsense mutation	c.950C>A	p.Ser317*	0.048
9	chr1	226,385,619	*PARP1*	Nonsense mutation	c.896C>A	p.Ser299*	0.041
9	chr9	99,138,006	*TGFBR1*	Nonsense mutation	c.734C>A	p.Ser245*	0.038
9	chr17	72,124,082	*SOX9*	Nonsense mutation	c.1225G>T	p.Glu409*	0.027
10	chr17	7,674,233	*TP53*	Missense mutation	c.730G>A	p.Gly244Ser	0.247
11	chr3	138,655,450	*PIK3CB*	Missense mutation	c.3152A>G	p.Glu1051Gly	0.210

11	chr7	129,205,643	*SMO*	Missense mutation	c.781C>T	p.Arg261Cys	0.029
11	chr9	77,922,196	*GNAQ*	Missense mutation	c.286A>T	p.Thr96Ser	0.116
12	chr3	179,234,297	*PIK3CA*	Missense mutation	c.3140A>T	p.His1047Leu	0.200
12	chr11	108,365,360	*ATM*	Missense mutation	c.9023G>A	p.Arg3008His	0.036
12	chr3	30,650,401	*TGFBR2*	Frameshift mutation	c.473delT	p.Phe158fs	0.256
12	chr7	152,235,876	*KMT2C*	Nonsense mutation	c.2710C>T	p.Arg904*	0.200
12	chr16	68,801,709	*CDH1*	Frameshift mutation	c.208delT	p.Ser70fs	0.112
12	chrX	40,072,420	*BCOR*	Nonsense mutation	c.2926C>T	p.Arg976*	0.122
13	chr1	26,729,726	*ARID1A*	Nonsense mutation	c.1213C>T	p.Gln405*	0.060
13	chr1	26,761,067	*ARID1A*	Nonsense mutation	c.2132C>G	p.Ser711*	0.100
13	chr3	179,218,294	*PIK3CA*	Missense mutation	c.1624G>A	p.Glu542Lys	0.132
13	chr5	150,124,275	*PDGFRB*	Missense mutation	c.1998C>G	p.Asn666Lys	0.074

14	chr3	179,218,304	*PIK3CA*	Missense mutation	c.1634A>C	p.Glu545Ala	0.093
14	chr1	26,780,532	*ARID1A*	Nonsense mutation	c.6634C>T	p.Gln2212*	0.126
14	chr10	87,957,872	*PTEN*	Nonsense mutation	c.1173C>A	p.Cys391*	0.054
14	chr10	87,957,915	*PTEN*	Nonsense mutation	c.1216C>T	p.Arg406*	0.029
14	chrX	40,062,895	*BCOR*	Nonsense mutation	c.4024C>T	p.Gln1342*	0.086
15	chr3	179,218,306	*PIK3CA*	Missense mutation	c.1636C>A	p.Gln546Lys	0.048
16	chr1	26,763,177	*ARID1A*	Frameshift mutation	c.2625_2628dupGCCA	p.Pro877fs	0.063
16	chr3	179,234,302	*PIK3CA*	Missense mutation	c.3145G>C	p.Gly1049Arg	0.032
17	chr1	26,729,739	*ARID1A*	Nonsense mutation	c.1226C>A	p.Ser409*	0.049
17	chr5	177,204,232	*NSD1*	Missense mutation	c.1176C>A	p.Phe392Leu	0.061
18	chr10	87,952,264	*PTEN*	Splice site mutation	c.1153+5G>A		0.192

18	chr1	26,696,659	*ARID1A*	Frameshift mutation	c.267_295delCAGCG GCGGCGGGCCCGG CGCGGAGCCGG	p.Ser90fs	0.098

Supplementary Table S4. Mutual exclusivity and co-occurrence of pathogenic genetic alterations in EBVaGCs

A	B	Neither	A Not B	B Not A	Both	Log2 Odds Ratio	p-Value	q-Value	Tendency
PD-L1	JAK2	15	0	0	3	>3	0.001	0.772	Co-occurrence
TP53	PD-L1	13	2	0	3	>3	0.012	0.944	Co-occurrence
TP53	JAK2	13	2	0	3	>3	0.012	0.944	Co-occurrence
PD-L1	PDCD1LG2	15	1	0	2	>3	0.02	0.944	Co-occurrence
JAK2	PDCD1LG2	15	1	0	2	>3	0.02	0.944	Co-occurrence
FBXW7	MUC6	14	2	0	2	>3	0.039	0.944	Co-occurrence
TP53	PIK3CA	6	5	7	0	<-3	0.054	0.944	Mutual exclusivity
PARP1	TGFBR1	17	0	0	1	>3	0.056	0.944	Co-occurrence

PARP1	SOX9	17	0	0	1	>3	0.056	0.944	Co-occurrence
TGFBR1	SOX9	17	0	0	1	>3	0.056	0.944	Co-occurrence
ERCC4	CIC	17	0	0	1	>3	0.056	0.944	Co-occurrence
ERCC4	SMARCB1	17	0	0	1	>3	0.056	0.944	Co-occurrence
CIC	SMARCB1	17	0	0	1	>3	0.056	0.944	Co-occurrence
PTPRT	RB1	17	0	0	1	>3	0.056	0.944	Co-occurrence
ATM	TGFBR2	17	0	0	1	>3	0.056	0.944	Co-occurrence
ATM	CDH1	17	0	0	1	>3	0.056	0.944	Co-occurrence
TGFBR2	CDH1	17	0	0	1	>3	0.056	0.944	Co-occurrence
SMO	GNAQ	17	0	0	1	>3	0.056	0.944	Co-occurrence
CBL	KMT2D	17	0	0	1	>3	0.056	0.944	Co-occurrence
TP53	PDCD1LG2	13	3	0	2	>3	0.065	0.944	Co-occurrence

PIK3CB	PTPRT	16	1	0	1	>3	0.111	0.944	Co-occurrence
KRAS	PTPRT	16	1	0	1	>3	0.111	0.944	Co-occurrence
PIK3CB	RB1	16	1	0	1	>3	0.111	0.944	Co-occurrence
KRAS	RB1	16	1	0	1	>3	0.111	0.944	Co-occurrence
KMT2C	ATM	16	1	0	1	>3	0.111	0.944	Co-occurrence
KMT2C	TGFBR2	16	1	0	1	>3	0.111	0.944	Co-occurrence
KMT2C	CDH1	16	1	0	1	>3	0.111	0.944	Co-occurrence
CTNNB1	EPHA3	16	1	0	1	>3	0.111	0.944	Co-occurrence
MUC6	EPHB1	16	1	0	1	>3	0.111	0.944	Co-occurrence
EPHB1	PDCD1LG2	16	0	1	1	>3	0.111	0.944	Co-occurrence
PIK3CB	SMO	16	1	0	1	>3	0.111	0.944	Co-occurrence
PIK3CB	GNAQ	16	1	0	1	>3	0.111	0.944	Co-occurrence

CTNNB1	CBL	16	1	0	1	>3	0.111	0.944	Co-occurrence
CTNNB1	KMT2D	16	1	0	1	>3	0.111	0.944	Co-occurrence
PIK3CA	KMT2C	11	5	0	2	>3	0.137	0.944	Co-occurrence
ERCC4	PD-L1	15	0	2	1	>3	0.167	0.944	Co-occurrence
CIC	PD-L1	15	0	2	1	>3	0.167	0.944	Co-occurrence
SMARCB1	PD-L1	15	0	2	1	>3	0.167	0.944	Co-occurrence
ERCC4	JAK2	15	0	2	1	>3	0.167	0.944	Co-occurrence
CIC	JAK2	15	0	2	1	>3	0.167	0.944	Co-occurrence
SMARCB1	JAK2	15	0	2	1	>3	0.167	0.944	Co-occurrence
ATM	BCOR	15	0	2	1	>3	0.167	0.944	Co-occurrence
TGFBR2	BCOR	15	0	2	1	>3	0.167	0.944	Co-occurrence
CDH1	BCOR	15	0	2	1	>3	0.167	0.944	Co-occurrence

PD-L1	EPHB1	15	2	0	1	>3	0.167	0.944	Co-occurrence
JAK2	EPHB1	15	2	0	1	>3	0.167	0.944	Co-occurrence
ERBB2	CBL	15	2	0	1	>3	0.167	0.944	Co-occurrence
BCOR	CBL	15	2	0	1	>3	0.167	0.944	Co-occurrence
ERBB2	KMT2D	15	2	0	1	>3	0.167	0.944	Co-occurrence
BCOR	KMT2D	15	2	0	1	>3	0.167	0.944	Co-occurrence
PIK3CA	ARID1A	7	2	4	5	2.129	0.167	0.944	Co-occurrence
PD-L1	PIK3CA	8	3	7	0	<-3	0.202	0.944	Mutual exclusivity
JAK2	PIK3CA	8	3	7	0	<-3	0.202	0.944	Mutual exclusivity
PIK3CB	KRAS	15	1	1	1	>3	0.216	0.944	Co-occurrence
KMT2C	MUC6	15	1	1	1	>3	0.216	0.944	Co-occurrence
MUC6	PDCD1LG2	15	1	1	1	>3	0.216	0.944	Co-occurrence

FBXW7	EPHA3	14	3	0	1	>3	0.222	0.944	Co-occurrence
FBXW7	EPHB1	14	3	0	1	>3	0.222	0.944	Co-occurrence
ARID1A	PIK3CB	7	9	2	0	<-3	0.235	0.944	Mutual exclusivity
ARID1A	MUC6	9	7	0	2	>3	0.235	0.944	Co-occurrence
ARID1A	CTNNB1	7	9	2	0	<-3	0.235	0.944	Mutual exclusivity
ARID1A	PTEN	9	7	0	2	>3	0.235	0.944	Co-occurrence
ERCC4	TP53	13	0	4	1	>3	0.278	0.944	Co-occurrence
TP53	CIC	13	4	0	1	>3	0.278	0.944	Co-occurrence
TP53	SMARCB1	13	4	0	1	>3	0.278	0.944	Co-occurrence
C1orf112	ATM	13	4	0	1	>3	0.278	0.944	Co-occurrence
C1orf112	TGFBR2	13	4	0	1	>3	0.278	0.944	Co-occurrence
C1orf112	CDH1	13	4	0	1	>3	0.278	0.944	Co-occurrence

TP53	EPHA3	13	4	0	1	>3	0.278	0.944	Co-occurrence
C1orf112	EPHA3	13	4	0	1	>3	0.278	0.944	Co-occurrence
TP53	EPHB1	13	4	0	1	>3	0.278	0.944	Co-occurrence
TP53	ZFHX3	13	4	0	1	>3	0.278	0.944	Co-occurrence
C1orf112	NSD1	13	4	0	1	>3	0.278	0.944	Co-occurrence
ARID1A	FBXW7	8	6	1	3	2	0.288	0.944	Co-occurrence
TP53	FBXW7	11	3	2	2	1.874	0.299	0.944	Co-occurrence
PD-L1	MUC6	14	2	1	1	2.807	0.314	0.944	Co-occurrence
JAK2	MUC6	14	2	1	1	2.807	0.314	0.944	Co-occurrence
KRAS	ERBB2	14	1	2	1	2.807	0.314	0.944	Co-occurrence
KMT2C	BCOR	14	1	2	1	2.807	0.314	0.944	Co-occurrence
ERBB2	CTNNB1	14	2	1	1	2.807	0.314	0.944	Co-occurrence

BCOR	CTNNB1	14	2	1	1	2.807	0.314	0.944	Co-occurrence
ERBB2	PDCD1LG2	14	2	1	1	2.807	0.314	0.944	Co-occurrence
BCOR	PTEN	14	2	1	1	2.807	0.314	0.944	Co-occurrence
PIK3CA	BCOR	10	5	1	2	2	0.326	0.944	Co-occurrence
PD-L1	C1orf112	10	3	5	0	<-3	0.35	0.944	Mutual exclusivity
JAK2	C1orf112	10	3	5	0	<-3	0.35	0.944	Mutual exclusivity
C1orf112	ERBB2	10	5	3	0	<-3	0.35	0.944	Mutual exclusivity
TP53	BCOR	10	5	3	0	<-3	0.35	0.944	Mutual exclusivity
PIK3CA	PIK3CB	9	7	2	0	<-3	0.359	0.944	Mutual exclusivity
PIK3CA	CTNNB1	9	7	2	0	<-3	0.359	0.944	Mutual exclusivity
PIK3CA	PDCD1LG2	9	7	2	0	<-3	0.359	0.944	Mutual exclusivity
PIK3CA	PDGFRB	11	6	0	1	>3	0.389	0.944	Co-occurrence

PIK3CA	ATM	11	6	0	1	>3	0.389	0.944	Co-occurrence
PIK3CA	TGFBR2	11	6	0	1	>3	0.389	0.944	Co-occurrence
PIK3CA	CDH1	11	6	0	1	>3	0.389	0.944	Co-occurrence
KRAS	FBXW7	13	1	3	1	2.115	0.405	0.944	Co-occurrence
FBXW7	KMT2C	13	3	1	1	2.115	0.405	0.944	Co-occurrence
FBXW7	CTNNB1	13	3	1	1	2.115	0.405	0.944	Co-occurrence
FBXW7	PDCD1LG2	13	3	1	1	2.115	0.405	0.944	Co-occurrence
PD-L1	ERBB2	13	2	2	1	1.7	0.442	0.944	Co-occurrence
JAK2	ERBB2	13	2	2	1	1.7	0.442	0.944	Co-occurrence
ERBB2	BCOR	13	2	2	1	1.7	0.442	0.944	Co-occurrence
FBXW7	BCOR	11	4	3	0	<-3	0.446	0.944	Mutual exclusivity
C1orf112	KMT2C	12	4	1	1	1.585	0.49	0.944	Co-occurrence

TP53	MUC6	12	4	1	1	1.585	0.49	0.944	Co-occurrence
TP53	CTNNB1	12	4	1	1	1.585	0.49	0.944	Co-occurrence
C1orf112	CTNNB1	12	4	1	1	1.585	0.49	0.944	Co-occurrence
C1orf112	PTEN	12	4	1	1	1.585	0.49	0.944	Co-occurrence
TP53	ARID1A	6	3	7	2	-0.807	0.5	0.944	Mutual exclusivity
C1orf112	ARID1A	6	3	7	2	-0.807	0.5	0.944	Mutual exclusivity
PARP1	ARID1A	8	1	9	0	<-3	0.5	0.944	Mutual exclusivity
TGFBR1	ARID1A	8	1	9	0	<-3	0.5	0.944	Mutual exclusivity
SOX9	ARID1A	8	1	9	0	<-3	0.5	0.944	Mutual exclusivity
ERCC4	ARID1A	8	1	9	0	<-3	0.5	0.944	Mutual exclusivity
CIC	ARID1A	8	1	9	0	<-3	0.5	0.944	Mutual exclusivity
SMARCB1	ARID1A	8	1	9	0	<-3	0.5	0.944	Mutual exclusivity

ARID1A	PDGFRB	9	8	0	1	>3	0.5	0.944	Co-occurrence
ARID1A	PTPRT	8	9	1	0	<-3	0.5	0.944	Mutual exclusivity
ARID1A	RB1	8	9	1	0	<-3	0.5	0.944	Mutual exclusivity
ARID1A	ATM	8	9	1	0	<-3	0.5	0.944	Mutual exclusivity
ARID1A	TGFBR2	8	9	1	0	<-3	0.5	0.944	Mutual exclusivity
ARID1A	CDH1	8	9	1	0	<-3	0.5	0.944	Mutual exclusivity
ARID1A	EPHA3	8	9	1	0	<-3	0.5	0.944	Mutual exclusivity
ARID1A	EPHB1	9	8	0	1	>3	0.5	0.944	Co-occurrence
ARID1A	SMO	8	9	1	0	<-3	0.5	0.944	Mutual exclusivity
ARID1A	GNAQ	8	9	1	0	<-3	0.5	0.944	Mutual exclusivity
ARID1A	CBL	8	9	1	0	<-3	0.5	0.944	Mutual exclusivity
ARID1A	KMT2D	8	9	1	0	<-3	0.5	0.944	Mutual exclusivity

ARID1A	ZFHX3	9	8	0	1	>3	0.5	0.944	Co-occurrence
ARID1A	NSD1	9	8	0	1	>3	0.5	0.944	Co-occurrence
ARID1A	ERBB2	7	8	2	1	-1.193	0.5	0.944	Mutual exclusivity
ARID1A	BCOR	7	8	2	1	-1.193	0.5	0.944	Mutual exclusivity
PD-L1	ARID1A	7	2	8	1	-1.193	0.5	0.944	Mutual exclusivity
JAK2	ARID1A	7	2	8	1	-1.193	0.5	0.944	Mutual exclusivity
TP53	PIK3CB	11	5	2	0	<-3	0.51	0.944	Mutual exclusivity
C1orf112	PIK3CB	11	5	2	0	<-3	0.51	0.944	Mutual exclusivity
TP53	KRAS	11	5	2	0	<-3	0.51	0.944	Mutual exclusivity
C1orf112	KRAS	11	5	2	0	<-3	0.51	0.944	Mutual exclusivity
TP53	KMT2C	11	5	2	0	<-3	0.51	0.944	Mutual exclusivity
C1orf112	MUC6	11	5	2	0	<-3	0.51	0.944	Mutual exclusivity

C1orf112	PDCD1LG2	11	5	2	0	<-3	0.51	0.944	Mutual exclusivity
TP53	PTEN	11	5	2	0	<-3	0.51	0.944	Mutual exclusivity
PIK3CA	FBXW7	9	5	2	2	0.848	0.515	0.944	Co-occurrence
PD-L1	FBXW7	12	2	3	1	1	0.554	0.944	Co-occurrence
JAK2	FBXW7	12	2	3	1	1	0.554	0.944	Co-occurrence
FBXW7	ERBB2	12	3	2	1	1	0.554	0.944	Co-occurrence
PD-L1	BCOR	12	3	3	0	<-3	0.558	0.944	Mutual exclusivity
JAK2	BCOR	12	3	3	0	<-3	0.558	0.944	Mutual exclusivity
TP53	C1orf112	9	4	4	1	-0.83	0.567	0.944	Mutual exclusivity
PIK3CB	FBXW7	12	2	4	0	<-3	0.595	0.944	Mutual exclusivity
FBXW7	PTEN	12	4	2	0	<-3	0.595	0.944	Mutual exclusivity
PARP1	PIK3CA	10	1	7	0	<-3	0.611	0.944	Mutual exclusivity

TGFBR1	PIK3CA	10	1	7	0	<-3	0.611	0.944	Mutual exclusivity
SOX9	PIK3CA	10	1	7	0	<-3	0.611	0.944	Mutual exclusivity
ERCC4	PIK3CA	10	1	7	0	<-3	0.611	0.944	Mutual exclusivity
CIC	PIK3CA	10	1	7	0	<-3	0.611	0.944	Mutual exclusivity
SMARCB1	PIK3CA	10	1	7	0	<-3	0.611	0.944	Mutual exclusivity
PIK3CA	PTPRT	10	7	1	0	<-3	0.611	0.944	Mutual exclusivity
PIK3CA	RB1	10	7	1	0	<-3	0.611	0.944	Mutual exclusivity
PIK3CA	EPHA3	10	7	1	0	<-3	0.611	0.944	Mutual exclusivity
PIK3CA	EPHB1	10	7	1	0	<-3	0.611	0.944	Mutual exclusivity
PIK3CA	SMO	10	7	1	0	<-3	0.611	0.944	Mutual exclusivity
PIK3CA	GNAQ	10	7	1	0	<-3	0.611	0.944	Mutual exclusivity
PIK3CA	CBL	10	7	1	0	<-3	0.611	0.944	Mutual exclusivity

PIK3CA	KMT2D	10	7	1	0	<-3	0.611	0.944	Mutual exclusivity
PIK3CA	ZFHX3	10	7	1	0	<-3	0.611	0.944	Mutual exclusivity
PIK3CA	NSD1	10	7	1	0	<-3	0.611	0.944	Mutual exclusivity
PIK3CA	KRAS	10	6	1	1	0.737	0.641	0.944	Co-occurrence
PIK3CA	MUC6	10	6	1	1	0.737	0.641	0.944	Co-occurrence
PIK3CA	PTEN	10	6	1	1	0.737	0.641	0.944	Co-occurrence
TP53	ERBB2	11	4	2	1	0.459	0.65	0.944	Co-occurrence
C1orf112	BCOR	11	4	2	1	0.459	0.65	0.944	Co-occurrence
PIK3CA	ERBB2	9	6	2	1	-0.415	0.674	0.944	Mutual exclusivity
PIK3CA	C1orf112	8	5	3	2	0.093	0.676	0.944	Co-occurrence
PD-L1	PIK3CB	13	3	2	0	<-3	0.686	0.944	Mutual exclusivity
JAK2	PIK3CB	13	3	2	0	<-3	0.686	0.944	Mutual exclusivity

PD-L1	KRAS	13	3	2	0	<-3	0.686	0.944	Mutual exclusivity
JAK2	KRAS	13	3	2	0	<-3	0.686	0.944	Mutual exclusivity
PD-L1	KMT2C	13	3	2	0	<-3	0.686	0.944	Mutual exclusivity
JAK2	KMT2C	13	3	2	0	<-3	0.686	0.944	Mutual exclusivity
PIK3CB	ERBB2	13	2	3	0	<-3	0.686	0.944	Mutual exclusivity
KMT2C	ERBB2	13	2	3	0	<-3	0.686	0.944	Mutual exclusivity
MUC6	ERBB2	13	2	3	0	<-3	0.686	0.944	Mutual exclusivity
PIK3CB	BCOR	13	2	3	0	<-3	0.686	0.944	Mutual exclusivity
KRAS	BCOR	13	2	3	0	<-3	0.686	0.944	Mutual exclusivity
MUC6	BCOR	13	2	3	0	<-3	0.686	0.944	Mutual exclusivity
PD-L1	CTNNB1	13	3	2	0	<-3	0.686	0.944	Mutual exclusivity
JAK2	CTNNB1	13	3	2	0	<-3	0.686	0.944	Mutual exclusivity

BCOR	PDCD1LG2	13	3	2	0	<-3	0.686	0.944	Mutual exclusivity
PD-L1	PTEN	13	3	2	0	<-3	0.686	0.944	Mutual exclusivity
JAK2	PTEN	13	3	2	0	<-3	0.686	0.944	Mutual exclusivity
ERBB2	PTEN	13	3	2	0	<-3	0.686	0.944	Mutual exclusivity
C1orf112	FBXW7	10	4	3	1	-0.263	0.701	0.944	Mutual exclusivity
PARP1	TP53	12	1	5	0	<-3	0.722	0.944	Mutual exclusivity
TGFBR1	TP53	12	1	5	0	<-3	0.722	0.944	Mutual exclusivity
SOX9	TP53	12	1	5	0	<-3	0.722	0.944	Mutual exclusivity
PARP1	C1orf112	12	1	5	0	<-3	0.722	0.944	Mutual exclusivity
TGFBR1	C1orf112	12	1	5	0	<-3	0.722	0.944	Mutual exclusivity
SOX9	C1orf112	12	1	5	0	<-3	0.722	0.944	Mutual exclusivity
ERCC4	C1orf112	12	1	5	0	<-3	0.722	0.944	Mutual exclusivity

CIC	C1orf112	12	1	5	0	<-3	0.722	0.944	Mutual exclusivity
SMARCB1	C1orf112	12	1	5	0	<-3	0.722	0.944	Mutual exclusivity
TP53	PDGFRB	12	5	1	0	<-3	0.722	0.944	Mutual exclusivity
C1orf112	PDGFRB	12	5	1	0	<-3	0.722	0.944	Mutual exclusivity
TP53	PTPRT	12	5	1	0	<-3	0.722	0.944	Mutual exclusivity
C1orf112	PTPRT	12	5	1	0	<-3	0.722	0.944	Mutual exclusivity
TP53	RB1	12	5	1	0	<-3	0.722	0.944	Mutual exclusivity
C1orf112	RB1	12	5	1	0	<-3	0.722	0.944	Mutual exclusivity
TP53	ATM	12	5	1	0	<-3	0.722	0.944	Mutual exclusivity
TP53	TGFBR2	12	5	1	0	<-3	0.722	0.944	Mutual exclusivity
TP53	CDH1	12	5	1	0	<-3	0.722	0.944	Mutual exclusivity
C1orf112	EPHB1	12	5	1	0	<-3	0.722	0.944	Mutual exclusivity

TP53	SMO	12	5	1	0	<-3	0.722	0.944	Mutual exclusivity
C1orf112	SMO	12	5	1	0	<-3	0.722	0.944	Mutual exclusivity
TP53	GNAQ	12	5	1	0	<-3	0.722	0.944	Mutual exclusivity
C1orf112	GNAQ	12	5	1	0	<-3	0.722	0.944	Mutual exclusivity
TP53	CBL	12	5	1	0	<-3	0.722	0.944	Mutual exclusivity
C1orf112	CBL	12	5	1	0	<-3	0.722	0.944	Mutual exclusivity
TP53	KMT2D	12	5	1	0	<-3	0.722	0.944	Mutual exclusivity
C1orf112	KMT2D	12	5	1	0	<-3	0.722	0.944	Mutual exclusivity
C1orf112	ZFHX3	12	5	1	0	<-3	0.722	0.944	Mutual exclusivity
TP53	NSD1	12	5	1	0	<-3	0.722	0.944	Mutual exclusivity
ARID1A	KRAS	8	8	1	1	0	0.765	0.944	Mutual exclusivity
ARID1A	KMT2C	8	8	1	1	0	0.765	0.944	Mutual exclusivity

ARID1A	PDCD1LG2	8	8	1	1	0	0.765	0.944	Mutual exclusivity
FBXW7	ATM	13	4	1	0	<-3	0.778	0.944	Mutual exclusivity
FBXW7	TGFBR2	13	4	1	0	<-3	0.778	0.944	Mutual exclusivity
FBXW7	CDH1	13	4	1	0	<-3	0.778	0.944	Mutual exclusivity
FBXW7	SMO	13	4	1	0	<-3	0.778	0.944	Mutual exclusivity
FBXW7	GNAQ	13	4	1	0	<-3	0.778	0.944	Mutual exclusivity
FBXW7	CBL	13	4	1	0	<-3	0.778	0.944	Mutual exclusivity
FBXW7	KMT2D	13	4	1	0	<-3	0.778	0.944	Mutual exclusivity
FBXW7	ZFHX3	13	4	1	0	<-3	0.778	0.944	Mutual exclusivity
FBXW7	NSD1	13	4	1	0	<-3	0.778	0.944	Mutual exclusivity
PARP1	FBXW7	13	1	4	0	<-3	0.778	0.944	Mutual exclusivity
TGFBR1	FBXW7	13	1	4	0	<-3	0.778	0.944	Mutual exclusivity

SOX9	FBXW7	13	1	4	0	<-3	0.778	0.944	Mutual exclusivity
ERCC4	FBXW7	13	1	4	0	<-3	0.778	0.944	Mutual exclusivity
CIC	FBXW7	13	1	4	0	<-3	0.778	0.944	Mutual exclusivity
SMARCB1	FBXW7	13	1	4	0	<-3	0.778	0.944	Mutual exclusivity
PDGFRB	FBXW7	13	1	4	0	<-3	0.778	0.944	Mutual exclusivity
PTPRT	FBXW7	13	1	4	0	<-3	0.778	0.944	Mutual exclusivity
RB1	FBXW7	13	1	4	0	<-3	0.778	0.944	Mutual exclusivity
PIK3CB	KMT2C	14	2	2	0	<-3	0.784	0.944	Mutual exclusivity
KRAS	KMT2C	14	2	2	0	<-3	0.784	0.944	Mutual exclusivity
PIK3CB	MUC6	14	2	2	0	<-3	0.784	0.944	Mutual exclusivity
KRAS	MUC6	14	2	2	0	<-3	0.784	0.944	Mutual exclusivity
PIK3CB	CTNNB1	14	2	2	0	<-3	0.784	0.944	Mutual exclusivity

KRAS	CTNNB1	14	2	2	0	<-3	0.784	0.944	Mutual exclusivity
KMT2C	CTNNB1	14	2	2	0	<-3	0.784	0.944	Mutual exclusivity
MUC6	CTNNB1	14	2	2	0	<-3	0.784	0.944	Mutual exclusivity
PIK3CB	PDCD1LG2	14	2	2	0	<-3	0.784	0.944	Mutual exclusivity
KRAS	PDCD1LG2	14	2	2	0	<-3	0.784	0.944	Mutual exclusivity
KMT2C	PDCD1LG2	14	2	2	0	<-3	0.784	0.944	Mutual exclusivity
CTNNB1	PDCD1LG2	14	2	2	0	<-3	0.784	0.944	Mutual exclusivity
PIK3CB	PTEN	14	2	2	0	<-3	0.784	0.944	Mutual exclusivity
KRAS	PTEN	14	2	2	0	<-3	0.784	0.944	Mutual exclusivity
KMT2C	PTEN	14	2	2	0	<-3	0.784	0.944	Mutual exclusivity
MUC6	PTEN	14	2	2	0	<-3	0.784	0.944	Mutual exclusivity
CTNNB1	PTEN	14	2	2	0	<-3	0.784	0.944	Mutual exclusivity

PDCD1LG2	PTEN	14	2	2	0	<-3	0.784	0.944	Mutual exclusivity
PARP1	PD-L1	14	1	3	0	<-3	0.833	0.944	Mutual exclusivity
TGFBR1	PD-L1	14	1	3	0	<-3	0.833	0.944	Mutual exclusivity
SOX9	PD-L1	14	1	3	0	<-3	0.833	0.944	Mutual exclusivity
PARP1	JAK2	14	1	3	0	<-3	0.833	0.944	Mutual exclusivity
TGFBR1	JAK2	14	1	3	0	<-3	0.833	0.944	Mutual exclusivity
SOX9	JAK2	14	1	3	0	<-3	0.833	0.944	Mutual exclusivity
PD-L1	PDGFRB	14	3	1	0	<-3	0.833	0.944	Mutual exclusivity
JAK2	PDGFRB	14	3	1	0	<-3	0.833	0.944	Mutual exclusivity
PD-L1	PTPRT	14	3	1	0	<-3	0.833	0.944	Mutual exclusivity
JAK2	PTPRT	14	3	1	0	<-3	0.833	0.944	Mutual exclusivity
PD-L1	RB1	14	3	1	0	<-3	0.833	0.944	Mutual exclusivity

JAK2	RB1	14	3	1	0	<-3	0.833	0.944	Mutual exclusivity
PARP1	ERBB2	14	1	3	0	<-3	0.833	0.944	Mutual exclusivity
TGFBR1	ERBB2	14	1	3	0	<-3	0.833	0.944	Mutual exclusivity
SOX9	ERBB2	14	1	3	0	<-3	0.833	0.944	Mutual exclusivity
ERCC4	ERBB2	14	1	3	0	<-3	0.833	0.944	Mutual exclusivity
CIC	ERBB2	14	1	3	0	<-3	0.833	0.944	Mutual exclusivity
SMARCB1	ERBB2	14	1	3	0	<-3	0.833	0.944	Mutual exclusivity
PDGFRB	ERBB2	14	1	3	0	<-3	0.833	0.944	Mutual exclusivity
PTPRT	ERBB2	14	1	3	0	<-3	0.833	0.944	Mutual exclusivity
RB1	ERBB2	14	1	3	0	<-3	0.833	0.944	Mutual exclusivity
PD-L1	ATM	14	3	1	0	<-3	0.833	0.944	Mutual exclusivity
JAK2	ATM	14	3	1	0	<-3	0.833	0.944	Mutual exclusivity

ERBB2	ATM	14	3	1	0	<-3	0.833	0.944	Mutual exclusivity
PD-L1	TGFBR2	14	3	1	0	<-3	0.833	0.944	Mutual exclusivity
JAK2	TGFBR2	14	3	1	0	<-3	0.833	0.944	Mutual exclusivity
ERBB2	TGFBR2	14	3	1	0	<-3	0.833	0.944	Mutual exclusivity
PD-L1	CDH1	14	3	1	0	<-3	0.833	0.944	Mutual exclusivity
JAK2	CDH1	14	3	1	0	<-3	0.833	0.944	Mutual exclusivity
ERBB2	CDH1	14	3	1	0	<-3	0.833	0.944	Mutual exclusivity
PARP1	BCOR	14	1	3	0	<-3	0.833	0.944	Mutual exclusivity
TGFBR1	BCOR	14	1	3	0	<-3	0.833	0.944	Mutual exclusivity
SOX9	BCOR	14	1	3	0	<-3	0.833	0.944	Mutual exclusivity
ERCC4	BCOR	14	1	3	0	<-3	0.833	0.944	Mutual exclusivity
CIC	BCOR	14	1	3	0	<-3	0.833	0.944	Mutual exclusivity

SMARCB1	BCOR	14	1	3	0	<-3	0.833	0.944	Mutual exclusivity
PDGFRB	BCOR	14	1	3	0	<-3	0.833	0.944	Mutual exclusivity
PTPRT	BCOR	14	1	3	0	<-3	0.833	0.944	Mutual exclusivity
RB1	BCOR	14	1	3	0	<-3	0.833	0.944	Mutual exclusivity
PD-L1	EPHA3	14	3	1	0	<-3	0.833	0.944	Mutual exclusivity
JAK2	EPHA3	14	3	1	0	<-3	0.833	0.944	Mutual exclusivity
ERBB2	EPHA3	14	3	1	0	<-3	0.833	0.944	Mutual exclusivity
BCOR	EPHA3	14	3	1	0	<-3	0.833	0.944	Mutual exclusivity
ERBB2	EPHB1	14	3	1	0	<-3	0.833	0.944	Mutual exclusivity
BCOR	EPHB1	14	3	1	0	<-3	0.833	0.944	Mutual exclusivity
PD-L1	SMO	14	3	1	0	<-3	0.833	0.944	Mutual exclusivity
JAK2	SMO	14	3	1	0	<-3	0.833	0.944	Mutual exclusivity

ERBB2	SMO	14	3	1	0	<-3	0.833	0.944	Mutual exclusivity
BCOR	SMO	14	3	1	0	<-3	0.833	0.944	Mutual exclusivity
PD-L1	GNAQ	14	3	1	0	<-3	0.833	0.944	Mutual exclusivity
JAK2	GNAQ	14	3	1	0	<-3	0.833	0.944	Mutual exclusivity
ERBB2	GNAQ	14	3	1	0	<-3	0.833	0.944	Mutual exclusivity
BCOR	GNAQ	14	3	1	0	<-3	0.833	0.944	Mutual exclusivity
PD-L1	CBL	14	3	1	0	<-3	0.833	0.944	Mutual exclusivity
JAK2	CBL	14	3	1	0	<-3	0.833	0.944	Mutual exclusivity
PD-L1	KMT2D	14	3	1	0	<-3	0.833	0.944	Mutual exclusivity
JAK2	KMT2D	14	3	1	0	<-3	0.833	0.944	Mutual exclusivity
PD-L1	ZFHX3	14	3	1	0	<-3	0.833	0.944	Mutual exclusivity
JAK2	ZFHX3	14	3	1	0	<-3	0.833	0.944	Mutual exclusivity

ERBB2	ZFHX3	14	3	1	0	<-3	0.833	0.944	Mutual exclusivity
BCOR	ZFHX3	14	3	1	0	<-3	0.833	0.944	Mutual exclusivity
PD-L1	NSD1	14	3	1	0	<-3	0.833	0.944	Mutual exclusivity
JAK2	NSD1	14	3	1	0	<-3	0.833	0.944	Mutual exclusivity
ERBB2	NSD1	14	3	1	0	<-3	0.833	0.944	Mutual exclusivity
BCOR	NSD1	14	3	1	0	<-3	0.833	0.944	Mutual exclusivity
PARP1	PIK3CB	15	1	2	0	<-3	0.889	0.944	Mutual exclusivity
TGFBR1	PIK3CB	15	1	2	0	<-3	0.889	0.944	Mutual exclusivity
SOX9	PIK3CB	15	1	2	0	<-3	0.889	0.944	Mutual exclusivity
ERCC4	PIK3CB	15	1	2	0	<-3	0.889	0.944	Mutual exclusivity
CIC	PIK3CB	15	1	2	0	<-3	0.889	0.944	Mutual exclusivity
SMARCB1	PIK3CB	15	1	2	0	<-3	0.889	0.944	Mutual exclusivity

PDGFRB	PIK3CB	15	1	2	0	<-3	0.889	0.944	Mutual exclusivity
PARP1	KRAS	15	1	2	0	<-3	0.889	0.944	Mutual exclusivity
TGFBR1	KRAS	15	1	2	0	<-3	0.889	0.944	Mutual exclusivity
SOX9	KRAS	15	1	2	0	<-3	0.889	0.944	Mutual exclusivity
ERCC4	KRAS	15	1	2	0	<-3	0.889	0.944	Mutual exclusivity
CIC	KRAS	15	1	2	0	<-3	0.889	0.944	Mutual exclusivity
SMARCB1	KRAS	15	1	2	0	<-3	0.889	0.944	Mutual exclusivity
PDGFRB	KRAS	15	1	2	0	<-3	0.889	0.944	Mutual exclusivity
PARP1	KMT2C	15	1	2	0	<-3	0.889	0.944	Mutual exclusivity
TGFBR1	KMT2C	15	1	2	0	<-3	0.889	0.944	Mutual exclusivity
SOX9	KMT2C	15	1	2	0	<-3	0.889	0.944	Mutual exclusivity
ERCC4	KMT2C	15	1	2	0	<-3	0.889	0.944	Mutual exclusivity

CIC	KMT2C	15	1	2	0	<-3	0.889	0.944	Mutual exclusivity
SMARCB1	KMT2C	15	1	2	0	<-3	0.889	0.944	Mutual exclusivity
PDGFRB	KMT2C	15	1	2	0	<-3	0.889	0.944	Mutual exclusivity
PTPRT	KMT2C	15	1	2	0	<-3	0.889	0.944	Mutual exclusivity
RB1	KMT2C	15	1	2	0	<-3	0.889	0.944	Mutual exclusivity
PARP1	MUC6	15	1	2	0	<-3	0.889	0.944	Mutual exclusivity
TGFBR1	MUC6	15	1	2	0	<-3	0.889	0.944	Mutual exclusivity
SOX9	MUC6	15	1	2	0	<-3	0.889	0.944	Mutual exclusivity
ERCC4	MUC6	15	1	2	0	<-3	0.889	0.944	Mutual exclusivity
CIC	MUC6	15	1	2	0	<-3	0.889	0.944	Mutual exclusivity
SMARCB1	MUC6	15	1	2	0	<-3	0.889	0.944	Mutual exclusivity
PDGFRB	MUC6	15	1	2	0	<-3	0.889	0.944	Mutual exclusivity

PTPRT	MUC6	15	1	2	0	<-3	0.889	0.944	Mutual exclusivity
RB1	MUC6	15	1	2	0	<-3	0.889	0.944	Mutual exclusivity
PIK3CB	ATM	15	2	1	0	<-3	0.889	0.944	Mutual exclusivity
KRAS	ATM	15	2	1	0	<-3	0.889	0.944	Mutual exclusivity
MUC6	ATM	15	2	1	0	<-3	0.889	0.944	Mutual exclusivity
PIK3CB	TGFBR2	15	2	1	0	<-3	0.889	0.944	Mutual exclusivity
KRAS	TGFBR2	15	2	1	0	<-3	0.889	0.944	Mutual exclusivity
MUC6	TGFBR2	15	2	1	0	<-3	0.889	0.944	Mutual exclusivity
PIK3CB	CDH1	15	2	1	0	<-3	0.889	0.944	Mutual exclusivity
KRAS	CDH1	15	2	1	0	<-3	0.889	0.944	Mutual exclusivity
MUC6	CDH1	15	2	1	0	<-3	0.889	0.944	Mutual exclusivity
PARP1	CTNNB1	15	1	2	0	<-3	0.889	0.944	Mutual exclusivity

TGFBR1	CTNNB1	15	1	2	0	<-3	0.889	0.944	Mutual exclusivity
SOX9	CTNNB1	15	1	2	0	<-3	0.889	0.944	Mutual exclusivity
ERCC4	CTNNB1	15	1	2	0	<-3	0.889	0.944	Mutual exclusivity
CIC	CTNNB1	15	1	2	0	<-3	0.889	0.944	Mutual exclusivity
SMARCB1	CTNNB1	15	1	2	0	<-3	0.889	0.944	Mutual exclusivity
PDGFRB	CTNNB1	15	1	2	0	<-3	0.889	0.944	Mutual exclusivity
PTPRT	CTNNB1	15	1	2	0	<-3	0.889	0.944	Mutual exclusivity
RB1	CTNNB1	15	1	2	0	<-3	0.889	0.944	Mutual exclusivity
ATM	CTNNB1	15	1	2	0	<-3	0.889	0.944	Mutual exclusivity
TGFBR2	CTNNB1	15	1	2	0	<-3	0.889	0.944	Mutual exclusivity
CDH1	CTNNB1	15	1	2	0	<-3	0.889	0.944	Mutual exclusivity
PIK3CB	EPHA3	15	2	1	0	<-3	0.889	0.944	Mutual exclusivity

KRAS	EPHA3	15	2	1	0	<-3	0.889	0.944	Mutual exclusivity
KMT2C	EPHA3	15	2	1	0	<-3	0.889	0.944	Mutual exclusivity
MUC6	EPHA3	15	2	1	0	<-3	0.889	0.944	Mutual exclusivity
PIK3CB	EPHB1	15	2	1	0	<-3	0.889	0.944	Mutual exclusivity
KRAS	EPHB1	15	2	1	0	<-3	0.889	0.944	Mutual exclusivity
KMT2C	EPHB1	15	2	1	0	<-3	0.889	0.944	Mutual exclusivity
CTNNB1	EPHB1	15	2	1	0	<-3	0.889	0.944	Mutual exclusivity
PARP1	PDCD1LG2	15	1	2	0	<-3	0.889	0.944	Mutual exclusivity
TGFBR1	PDCD1LG2	15	1	2	0	<-3	0.889	0.944	Mutual exclusivity
SOX9	PDCD1LG2	15	1	2	0	<-3	0.889	0.944	Mutual exclusivity
ERCC4	PDCD1LG2	15	1	2	0	<-3	0.889	0.944	Mutual exclusivity
CIC	PDCD1LG2	15	1	2	0	<-3	0.889	0.944	Mutual exclusivity

SMARCB1	PDCD1LG2	15	1	2	0	<-3	0.889	0.944	Mutual exclusivity
PDGFRB	PDCD1LG2	15	1	2	0	<-3	0.889	0.944	Mutual exclusivity
PTPRT	PDCD1LG2	15	1	2	0	<-3	0.889	0.944	Mutual exclusivity
RB1	PDCD1LG2	15	1	2	0	<-3	0.889	0.944	Mutual exclusivity
ATM	PDCD1LG2	15	1	2	0	<-3	0.889	0.944	Mutual exclusivity
TGFBR2	PDCD1LG2	15	1	2	0	<-3	0.889	0.944	Mutual exclusivity
CDH1	PDCD1LG2	15	1	2	0	<-3	0.889	0.944	Mutual exclusivity
EPHA3	PDCD1LG2	15	1	2	0	<-3	0.889	0.944	Mutual exclusivity
KRAS	SMO	15	2	1	0	<-3	0.889	0.944	Mutual exclusivity
KMT2C	SMO	15	2	1	0	<-3	0.889	0.944	Mutual exclusivity
MUC6	SMO	15	2	1	0	<-3	0.889	0.944	Mutual exclusivity
CTNNB1	SMO	15	2	1	0	<-3	0.889	0.944	Mutual exclusivity

PDCD1LG2	SMO	15	2	1	0	<-3	0.889	0.944	Mutual exclusivity
KRAS	GNAQ	15	2	1	0	<-3	0.889	0.944	Mutual exclusivity
KMT2C	GNAQ	15	2	1	0	<-3	0.889	0.944	Mutual exclusivity
MUC6	GNAQ	15	2	1	0	<-3	0.889	0.944	Mutual exclusivity
CTNNB1	GNAQ	15	2	1	0	<-3	0.889	0.944	Mutual exclusivity
PDCD1LG2	GNAQ	15	2	1	0	<-3	0.889	0.944	Mutual exclusivity
PIK3CB	CBL	15	2	1	0	<-3	0.889	0.944	Mutual exclusivity
KRAS	CBL	15	2	1	0	<-3	0.889	0.944	Mutual exclusivity
KMT2C	CBL	15	2	1	0	<-3	0.889	0.944	Mutual exclusivity
MUC6	CBL	15	2	1	0	<-3	0.889	0.944	Mutual exclusivity
PDCD1LG2	CBL	15	2	1	0	<-3	0.889	0.944	Mutual exclusivity
PIK3CB	KMT2D	15	2	1	0	<-3	0.889	0.944	Mutual exclusivity

KRAS	KMT2D	15	2	1	0	<-3	0.889	0.944	Mutual exclusivity
KMT2C	KMT2D	15	2	1	0	<-3	0.889	0.944	Mutual exclusivity
MUC6	KMT2D	15	2	1	0	<-3	0.889	0.944	Mutual exclusivity
PDCD1LG2	KMT2D	15	2	1	0	<-3	0.889	0.944	Mutual exclusivity
PARP1	PTEN	15	1	2	0	<-3	0.889	0.944	Mutual exclusivity
TGFBR1	PTEN	15	1	2	0	<-3	0.889	0.944	Mutual exclusivity
SOX9	PTEN	15	1	2	0	<-3	0.889	0.944	Mutual exclusivity
ERCC4	PTEN	15	1	2	0	<-3	0.889	0.944	Mutual exclusivity
CIC	PTEN	15	1	2	0	<-3	0.889	0.944	Mutual exclusivity
SMARCB1	PTEN	15	1	2	0	<-3	0.889	0.944	Mutual exclusivity
PDGFRB	PTEN	15	1	2	0	<-3	0.889	0.944	Mutual exclusivity
PTPRT	PTEN	15	1	2	0	<-3	0.889	0.944	Mutual exclusivity

RB1	PTEN	15	1	2	0	<-3	0.889	0.944	Mutual exclusivity
ATM	PTEN	15	1	2	0	<-3	0.889	0.944	Mutual exclusivity
TGFBR2	PTEN	15	1	2	0	<-3	0.889	0.944	Mutual exclusivity
CDH1	PTEN	15	1	2	0	<-3	0.889	0.944	Mutual exclusivity
EPHA3	PTEN	15	1	2	0	<-3	0.889	0.944	Mutual exclusivity
EPHB1	PTEN	15	1	2	0	<-3	0.889	0.944	Mutual exclusivity
SMO	PTEN	15	1	2	0	<-3	0.889	0.944	Mutual exclusivity
GNAQ	PTEN	15	1	2	0	<-3	0.889	0.944	Mutual exclusivity
CBL	PTEN	15	1	2	0	<-3	0.889	0.944	Mutual exclusivity
KMT2D	PTEN	15	1	2	0	<-3	0.889	0.944	Mutual exclusivity
PIK3CB	ZFHX3	15	2	1	0	<-3	0.889	0.944	Mutual exclusivity
KRAS	ZFHX3	15	2	1	0	<-3	0.889	0.944	Mutual exclusivity

KMT2C	ZFHX3	15	2	1	0	<-3	0.889	0.944	Mutual exclusivity
MUC6	ZFHX3	15	2	1	0	<-3	0.889	0.944	Mutual exclusivity
CTNNB1	ZFHX3	15	2	1	0	<-3	0.889	0.944	Mutual exclusivity
PDCD1LG2	ZFHX3	15	2	1	0	<-3	0.889	0.944	Mutual exclusivity
PTEN	ZFHX3	15	2	1	0	<-3	0.889	0.944	Mutual exclusivity
PIK3CB	NSD1	15	2	1	0	<-3	0.889	0.944	Mutual exclusivity
KRAS	NSD1	15	2	1	0	<-3	0.889	0.944	Mutual exclusivity
KMT2C	NSD1	15	2	1	0	<-3	0.889	0.944	Mutual exclusivity
MUC6	NSD1	15	2	1	0	<-3	0.889	0.944	Mutual exclusivity
CTNNB1	NSD1	15	2	1	0	<-3	0.889	0.944	Mutual exclusivity
PDCD1LG2	NSD1	15	2	1	0	<-3	0.889	0.944	Mutual exclusivity
PTEN	NSD1	15	2	1	0	<-3	0.889	0.944	Mutual exclusivity

PARP1	ERCC4	16	1	1	0	<-3	0.944	0.944	Mutual exclusivity
TGFBR1	ERCC4	16	1	1	0	<-3	0.944	0.944	Mutual exclusivity
SOX9	ERCC4	16	1	1	0	<-3	0.944	0.944	Mutual exclusivity
PARP1	CIC	16	1	1	0	<-3	0.944	0.944	Mutual exclusivity
TGFBR1	CIC	16	1	1	0	<-3	0.944	0.944	Mutual exclusivity
SOX9	CIC	16	1	1	0	<-3	0.944	0.944	Mutual exclusivity
PARP1	SMARCB1	16	1	1	0	<-3	0.944	0.944	Mutual exclusivity
TGFBR1	SMARCB1	16	1	1	0	<-3	0.944	0.944	Mutual exclusivity
SOX9	SMARCB1	16	1	1	0	<-3	0.944	0.944	Mutual exclusivity
PARP1	PDGFRB	16	1	1	0	<-3	0.944	0.944	Mutual exclusivity
TGFBR1	PDGFRB	16	1	1	0	<-3	0.944	0.944	Mutual exclusivity
SOX9	PDGFRB	16	1	1	0	<-3	0.944	0.944	Mutual exclusivity

ERCC4	PDGFRB	16	1	1	0	<-3	0.944	0.944	Mutual exclusivity
CIC	PDGFRB	16	1	1	0	<-3	0.944	0.944	Mutual exclusivity
SMARCB1	PDGFRB	16	1	1	0	<-3	0.944	0.944	Mutual exclusivity
PARP1	PTPRT	16	1	1	0	<-3	0.944	0.944	Mutual exclusivity
TGFBR1	PTPRT	16	1	1	0	<-3	0.944	0.944	Mutual exclusivity
SOX9	PTPRT	16	1	1	0	<-3	0.944	0.944	Mutual exclusivity
ERCC4	PTPRT	16	1	1	0	<-3	0.944	0.944	Mutual exclusivity
CIC	PTPRT	16	1	1	0	<-3	0.944	0.944	Mutual exclusivity
SMARCB1	PTPRT	16	1	1	0	<-3	0.944	0.944	Mutual exclusivity
PDGFRB	PTPRT	16	1	1	0	<-3	0.944	0.944	Mutual exclusivity
PARP1	RB1	16	1	1	0	<-3	0.944	0.944	Mutual exclusivity
TGFBR1	RB1	16	1	1	0	<-3	0.944	0.944	Mutual exclusivity

SOX9	RB1	16	1	1	0	<-3	0.944	0.944	Mutual exclusivity
ERCC4	RB1	16	1	1	0	<-3	0.944	0.944	Mutual exclusivity
CIC	RB1	16	1	1	0	<-3	0.944	0.944	Mutual exclusivity
SMARCB1	RB1	16	1	1	0	<-3	0.944	0.944	Mutual exclusivity
PDGFRB	RB1	16	1	1	0	<-3	0.944	0.944	Mutual exclusivity
PARP1	ATM	16	1	1	0	<-3	0.944	0.944	Mutual exclusivity
TGFBR1	ATM	16	1	1	0	<-3	0.944	0.944	Mutual exclusivity
SOX9	ATM	16	1	1	0	<-3	0.944	0.944	Mutual exclusivity
ERCC4	ATM	16	1	1	0	<-3	0.944	0.944	Mutual exclusivity
CIC	ATM	16	1	1	0	<-3	0.944	0.944	Mutual exclusivity
SMARCB1	ATM	16	1	1	0	<-3	0.944	0.944	Mutual exclusivity
PDGFRB	ATM	16	1	1	0	<-3	0.944	0.944	Mutual exclusivity

PTPRT	ATM	16	1	1	0	<-3	0.944	0.944	Mutual exclusivity
RB1	ATM	16	1	1	0	<-3	0.944	0.944	Mutual exclusivity
PARP1	TGFBR2	16	1	1	0	<-3	0.944	0.944	Mutual exclusivity
TGFBR1	TGFBR2	16	1	1	0	<-3	0.944	0.944	Mutual exclusivity
SOX9	TGFBR2	16	1	1	0	<-3	0.944	0.944	Mutual exclusivity
ERCC4	TGFBR2	16	1	1	0	<-3	0.944	0.944	Mutual exclusivity
CIC	TGFBR2	16	1	1	0	<-3	0.944	0.944	Mutual exclusivity
SMARCB1	TGFBR2	16	1	1	0	<-3	0.944	0.944	Mutual exclusivity
PDGFRB	TGFBR2	16	1	1	0	<-3	0.944	0.944	Mutual exclusivity
PTPRT	TGFBR2	16	1	1	0	<-3	0.944	0.944	Mutual exclusivity
RB1	TGFBR2	16	1	1	0	<-3	0.944	0.944	Mutual exclusivity
PARP1	CDH1	16	1	1	0	<-3	0.944	0.944	Mutual exclusivity

TGFBR1	CDH1	16	1	1	0	<-3	0.944	0.944	Mutual exclusivity
SOX9	CDH1	16	1	1	0	<-3	0.944	0.944	Mutual exclusivity
ERCC4	CDH1	16	1	1	0	<-3	0.944	0.944	Mutual exclusivity
CIC	CDH1	16	1	1	0	<-3	0.944	0.944	Mutual exclusivity
SMARCB1	CDH1	16	1	1	0	<-3	0.944	0.944	Mutual exclusivity
PDGFRB	CDH1	16	1	1	0	<-3	0.944	0.944	Mutual exclusivity
PTPRT	CDH1	16	1	1	0	<-3	0.944	0.944	Mutual exclusivity
RB1	CDH1	16	1	1	0	<-3	0.944	0.944	Mutual exclusivity
PARP1	EPHA3	16	1	1	0	<-3	0.944	0.944	Mutual exclusivity
TGFBR1	EPHA3	16	1	1	0	<-3	0.944	0.944	Mutual exclusivity
SOX9	EPHA3	16	1	1	0	<-3	0.944	0.944	Mutual exclusivity
ERCC4	EPHA3	16	1	1	0	<-3	0.944	0.944	Mutual exclusivity

CIC	EPHA3	16	1	1	0	<-3	0.944	0.944	Mutual exclusivity
SMARCB1	EPHA3	16	1	1	0	<-3	0.944	0.944	Mutual exclusivity
PDGFRB	EPHA3	16	1	1	0	<-3	0.944	0.944	Mutual exclusivity
PTPRT	EPHA3	16	1	1	0	<-3	0.944	0.944	Mutual exclusivity
RB1	EPHA3	16	1	1	0	<-3	0.944	0.944	Mutual exclusivity
ATM	EPHA3	16	1	1	0	<-3	0.944	0.944	Mutual exclusivity
TGFBR2	EPHA3	16	1	1	0	<-3	0.944	0.944	Mutual exclusivity
CDH1	EPHA3	16	1	1	0	<-3	0.944	0.944	Mutual exclusivity
PARP1	EPHB1	16	1	1	0	<-3	0.944	0.944	Mutual exclusivity
TGFBR1	EPHB1	16	1	1	0	<-3	0.944	0.944	Mutual exclusivity
SOX9	EPHB1	16	1	1	0	<-3	0.944	0.944	Mutual exclusivity
ERCC4	EPHB1	16	1	1	0	<-3	0.944	0.944	Mutual exclusivity

CIC	EPHB1	16	1	1	0	<-3	0.944	0.944	Mutual exclusivity
SMARCB1	EPHB1	16	1	1	0	<-3	0.944	0.944	Mutual exclusivity
PDGFRB	EPHB1	16	1	1	0	<-3	0.944	0.944	Mutual exclusivity
PTPRT	EPHB1	16	1	1	0	<-3	0.944	0.944	Mutual exclusivity
RB1	EPHB1	16	1	1	0	<-3	0.944	0.944	Mutual exclusivity
ATM	EPHB1	16	1	1	0	<-3	0.944	0.944	Mutual exclusivity
TGFBR2	EPHB1	16	1	1	0	<-3	0.944	0.944	Mutual exclusivity
CDH1	EPHB1	16	1	1	0	<-3	0.944	0.944	Mutual exclusivity
EPHA3	EPHB1	16	1	1	0	<-3	0.944	0.944	Mutual exclusivity
PARP1	SMO	16	1	1	0	<-3	0.944	0.944	Mutual exclusivity
TGFBR1	SMO	16	1	1	0	<-3	0.944	0.944	Mutual exclusivity
SOX9	SMO	16	1	1	0	<-3	0.944	0.944	Mutual exclusivity

ERCC4	SMO	16	1	1	0	<-3	0.944	0.944	Mutual exclusivity
CIC	SMO	16	1	1	0	<-3	0.944	0.944	Mutual exclusivity
SMARCB1	SMO	16	1	1	0	<-3	0.944	0.944	Mutual exclusivity
PDGFRB	SMO	16	1	1	0	<-3	0.944	0.944	Mutual exclusivity
PTPRT	SMO	16	1	1	0	<-3	0.944	0.944	Mutual exclusivity
RB1	SMO	16	1	1	0	<-3	0.944	0.944	Mutual exclusivity
ATM	SMO	16	1	1	0	<-3	0.944	0.944	Mutual exclusivity
TGFBR2	SMO	16	1	1	0	<-3	0.944	0.944	Mutual exclusivity
CDH1	SMO	16	1	1	0	<-3	0.944	0.944	Mutual exclusivity
EPHA3	SMO	16	1	1	0	<-3	0.944	0.944	Mutual exclusivity
EPHB1	SMO	16	1	1	0	<-3	0.944	0.944	Mutual exclusivity
PARP1	GNAQ	16	1	1	0	<-3	0.944	0.944	Mutual exclusivity

TGFBR1	GNAQ	16	1	1	0	<-3	0.944	0.944	Mutual exclusivity
SOX9	GNAQ	16	1	1	0	<-3	0.944	0.944	Mutual exclusivity
ERCC4	GNAQ	16	1	1	0	<-3	0.944	0.944	Mutual exclusivity
CIC	GNAQ	16	1	1	0	<-3	0.944	0.944	Mutual exclusivity
SMARCB1	GNAQ	16	1	1	0	<-3	0.944	0.944	Mutual exclusivity
PDGFRB	GNAQ	16	1	1	0	<-3	0.944	0.944	Mutual exclusivity
PTPRT	GNAQ	16	1	1	0	<-3	0.944	0.944	Mutual exclusivity
RB1	GNAQ	16	1	1	0	<-3	0.944	0.944	Mutual exclusivity
ATM	GNAQ	16	1	1	0	<-3	0.944	0.944	Mutual exclusivity
TGFBR2	GNAQ	16	1	1	0	<-3	0.944	0.944	Mutual exclusivity
CDH1	GNAQ	16	1	1	0	<-3	0.944	0.944	Mutual exclusivity
EPHA3	GNAQ	16	1	1	0	<-3	0.944	0.944	Mutual exclusivity

EPHB1	GNAQ	16	1	1	0	<-3	0.944	0.944	Mutual exclusivity
PARP1	CBL	16	1	1	0	<-3	0.944	0.944	Mutual exclusivity
TGFBR1	CBL	16	1	1	0	<-3	0.944	0.944	Mutual exclusivity
SOX9	CBL	16	1	1	0	<-3	0.944	0.944	Mutual exclusivity
ERCC4	CBL	16	1	1	0	<-3	0.944	0.944	Mutual exclusivity
CIC	CBL	16	1	1	0	<-3	0.944	0.944	Mutual exclusivity
SMARCB1	CBL	16	1	1	0	<-3	0.944	0.944	Mutual exclusivity
PDGFRB	CBL	16	1	1	0	<-3	0.944	0.944	Mutual exclusivity
PTPRT	CBL	16	1	1	0	<-3	0.944	0.944	Mutual exclusivity
RB1	CBL	16	1	1	0	<-3	0.944	0.944	Mutual exclusivity
ATM	CBL	16	1	1	0	<-3	0.944	0.944	Mutual exclusivity
TGFBR2	CBL	16	1	1	0	<-3	0.944	0.944	Mutual exclusivity

CDH1	CBL	16	1	1	0	<-3	0.944	0.944	Mutual exclusivity
EPHA3	CBL	16	1	1	0	<-3	0.944	0.944	Mutual exclusivity
EPHB1	CBL	16	1	1	0	<-3	0.944	0.944	Mutual exclusivity
SMO	CBL	16	1	1	0	<-3	0.944	0.944	Mutual exclusivity
GNAQ	CBL	16	1	1	0	<-3	0.944	0.944	Mutual exclusivity
PARP1	KMT2D	16	1	1	0	<-3	0.944	0.944	Mutual exclusivity
TGFBR1	KMT2D	16	1	1	0	<-3	0.944	0.944	Mutual exclusivity
SOX9	KMT2D	16	1	1	0	<-3	0.944	0.944	Mutual exclusivity
ERCC4	KMT2D	16	1	1	0	<-3	0.944	0.944	Mutual exclusivity
CIC	KMT2D	16	1	1	0	<-3	0.944	0.944	Mutual exclusivity
SMARCB1	KMT2D	16	1	1	0	<-3	0.944	0.944	Mutual exclusivity
PDGFRB	KMT2D	16	1	1	0	<-3	0.944	0.944	Mutual exclusivity

PTPRT	KMT2D	16	1	1	0	<-3	0.944	0.944	Mutual exclusivity
RB1	KMT2D	16	1	1	0	<-3	0.944	0.944	Mutual exclusivity
ATM	KMT2D	16	1	1	0	<-3	0.944	0.944	Mutual exclusivity
TGFBR2	KMT2D	16	1	1	0	<-3	0.944	0.944	Mutual exclusivity
CDH1	KMT2D	16	1	1	0	<-3	0.944	0.944	Mutual exclusivity
EPHA3	KMT2D	16	1	1	0	<-3	0.944	0.944	Mutual exclusivity
EPHB1	KMT2D	16	1	1	0	<-3	0.944	0.944	Mutual exclusivity
SMO	KMT2D	16	1	1	0	<-3	0.944	0.944	Mutual exclusivity
GNAQ	KMT2D	16	1	1	0	<-3	0.944	0.944	Mutual exclusivity
PARP1	ZFHX3	16	1	1	0	<-3	0.944	0.944	Mutual exclusivity
TGFBR1	ZFHX3	16	1	1	0	<-3	0.944	0.944	Mutual exclusivity
SOX9	ZFHX3	16	1	1	0	<-3	0.944	0.944	Mutual exclusivity

ERCC4	ZFHX3	16	1	1	0	<-3	0.944	0.944	Mutual exclusivity
CIC	ZFHX3	16	1	1	0	<-3	0.944	0.944	Mutual exclusivity
SMARCB1	ZFHX3	16	1	1	0	<-3	0.944	0.944	Mutual exclusivity
PDGFRB	ZFHX3	16	1	1	0	<-3	0.944	0.944	Mutual exclusivity
PTPRT	ZFHX3	16	1	1	0	<-3	0.944	0.944	Mutual exclusivity
RB1	ZFHX3	16	1	1	0	<-3	0.944	0.944	Mutual exclusivity
ATM	ZFHX3	16	1	1	0	<-3	0.944	0.944	Mutual exclusivity
TGFBR2	ZFHX3	16	1	1	0	<-3	0.944	0.944	Mutual exclusivity
CDH1	ZFHX3	16	1	1	0	<-3	0.944	0.944	Mutual exclusivity
EPHA3	ZFHX3	16	1	1	0	<-3	0.944	0.944	Mutual exclusivity
EPHB1	ZFHX3	16	1	1	0	<-3	0.944	0.944	Mutual exclusivity
SMO	ZFHX3	16	1	1	0	<-3	0.944	0.944	Mutual exclusivity

GNAQ	ZFHX3	16	1	1	0	<-3	0.944	0.944	Mutual exclusivity
CBL	ZFHX3	16	1	1	0	<-3	0.944	0.944	Mutual exclusivity
KMT2D	ZFHX3	16	1	1	0	<-3	0.944	0.944	Mutual exclusivity
PARP1	NSD1	16	1	1	0	<-3	0.944	0.944	Mutual exclusivity
TGFBR1	NSD1	16	1	1	0	<-3	0.944	0.944	Mutual exclusivity
SOX9	NSD1	16	1	1	0	<-3	0.944	0.944	Mutual exclusivity
ERCC4	NSD1	16	1	1	0	<-3	0.944	0.944	Mutual exclusivity
CIC	NSD1	16	1	1	0	<-3	0.944	0.944	Mutual exclusivity
SMARCB1	NSD1	16	1	1	0	<-3	0.944	0.944	Mutual exclusivity
PDGFRB	NSD1	16	1	1	0	<-3	0.944	0.944	Mutual exclusivity
PTPRT	NSD1	16	1	1	0	<-3	0.944	0.944	Mutual exclusivity
RB1	NSD1	16	1	1	0	<-3	0.944	0.944	Mutual exclusivity

ATM	NSD1	16	1	1	0	<-3	0.944	0.944	Mutual exclusivity
TGFBR2	NSD1	16	1	1	0	<-3	0.944	0.944	Mutual exclusivity
CDH1	NSD1	16	1	1	0	<-3	0.944	0.944	Mutual exclusivity
EPHA3	NSD1	16	1	1	0	<-3	0.944	0.944	Mutual exclusivity
EPHB1	NSD1	16	1	1	0	<-3	0.944	0.944	Mutual exclusivity
SMO	NSD1	16	1	1	0	<-3	0.944	0.944	Mutual exclusivity
GNAQ	NSD1	16	1	1	0	<-3	0.944	0.944	Mutual exclusivity
CBL	NSD1	16	1	1	0	<-3	0.944	0.944	Mutual exclusivity
KMT2D	NSD1	16	1	1	0	<-3	0.944	0.944	Mutual exclusivity
ZFHX3	NSD1	16	1	1	0	<-3	0.944	0.944	Mutual exclusivity

Table S5. Clinocopathologic characteristics of EBVaGCs accroding to degree of TILs

Variables		TIL Low (n=5)	TIL High (n=13)	p-value
Predominant histology	Intestinal-type like	2 (40.0%)	8 (61.5%)	0.007
	GCLS	0 (0.0%)	5 (38.5%)	
	PCC	3 (60.0%)	0 (0.0%)	
TPS (mean±SD)		1.0±2.2	25.7±37.0	0.034
TPS	≥1%	1 (20.0%)	8 (61.5%)	0.293
	<1%	4 (80.0%)	5 (38.5%)	
CPS		4.6±8.7	48.7±37.6	0.001
CPS	≥1	2 (40.0%)	13 (100.0%)	0.019
	<1	3 (60.0%)	0 (0.0%)	
TP53 mutation	Present	0 (0.0%)	5 (38.5%)	0.296
	Absent	5 (100.0%)	8 (61.5%)	
Size of tumor		7.1±3.1	3.9±2.2	0.025

Lymphatic invasion	Present	3 (60.0%)	5 (38.5%)	0.769
	Absent	2 (40.0%)	8 (61.5%)	
Vascular invasion	Present	2 (40.0%)	0 (0.0%)	0.114
	Absent	3 (60.0%)	13 (100.0%)	
PNI	Present	3 (60.0%)	4 (30.8%)	0.549
	Absent	2 (40.0%)	9 (69.2%)	
Stage	I	0 (0.0%)	7 (53.8%)	0.107
	II	2 (40.0%)	2 (15.4%)	
	III	3 (60.0%)	4 (30.8%)	
Age, years		64.2±6.1	65.2±5.4	0.729
Sex	Male	4 (80.0%)	11 (84.6%)	1.000
	Female	1 (20.0%)	2 (15.4%)	
LN metastasis	Present	3 (60.0%)	5 (38.5%)	0.769
	Absent	2 (40.0%)	8 (61.5%)	

TIL, tumor-infiltrating lymphocyte; GCLS, gastric carcinoma with lymphoid stroma; PCC, poorly cohesive carcinoma; SD, standard deviation; TPS, tumor proportion score; CPS,

combined positive score; TP53, tumor protein p53; PNI, perineural invasion; LN, lymph node

Table S6. TIL percentage and pathogenic alterations of EBVaGCs according to histologic pattern (TCGA cohort)

PathID	Histology review	TIL (%)	Pathogenic genetic mutation	Copy number alteration
TCGA-B7-5818-01	Intestinal	50	*PIK3CA*, *ARID1A*, *TP53*	
TCGA-BR-4253-01	Intestinal	60	*PIK3CA*, *ARID1A*, *PTEN*, *CD58*	
TCGA-BR-6455-01	Intestinal	15	*NRAS*, *PIK3CA*, *KMT2D*, *ARID2*, *BCOR*	*PTEN* (del)
TCGA-BR-6706-01	Intestinal	15	*PIK3CA*, *CDK12*, *ERBB3*, *MAX*, *PTPRT*	*EGFR* (amp) *JAK2* (amp), *CD274* (amp), *PDCD1LG2* (amp)
TCGA-BR-6707-01	Intestinal	15	*PIK3CA*, *PTEN*, *ARID1A*, *BCOR*	
TCGA-BR-7958-01	Intestinal	30	*SMAD3*	*PIK3CA* (amp), *PIK3CB* (amp)
TCGA-BR-8285-01	Intestinal	25		*PTEN* (del), *JAK2* (amp), *CD274* (amp), *PDCD1LG2* (amp)

TCGA-BR-8381-01	Intestinal	60	*PTEN*, *ARID1A*, *KMT2D*	*CD274* (amp), *PDCD1LG2* (amp), *JAK2* (amp)
TCGA-BR-8589-01	Intestinal	65	*ARID1A*, *CTNNB1*, *MGA*, *SETD2*, *RAD17*	
TCGA-BR-8676-01	Intestinal	20	*PIK3CA*, *KMT2C*, *KMT2D*	*FBXW7* (del), *PIK3CA* (amp)
TCGA-CD-5801-01	Intestinal	60	*BRAF*, *PIK3CA*, *MAP2K2*, *EPHA3*	*RICTOR* (amp)
TCGA-D7-5577-01	Intestinal	5	*MAP2K1*, *PIK3CA*, *PTEN*, *ARID1A*	
TCGA-D7-8570-01	Intestinal	50	*B2M*	*RICTOR* (amp), *TERT* (amp), *IGF1R* (amp)
TCGA-D7-8573-01	Intestinal	10	*CTNNB1*	*ARID1B* (del)
TCGA-D7-A4YX-01	Intestinal	40	*PIK3CA*, *ARID1A*	*ERBB2* (amp)
TCGA-FP-7916-01	Intestinal	25	*PIK3CA*, *ARID1A*	

TCGA-FP-7998-01	Intestinal	70	*PIK3CA*, *ARID1A*, *STAT3*	*JAK2* (amp), *CD274* (amp), *MDM2* (amp), *AJUBA* (del)
TCGA-HU-A4G6-01	Intestinal	N/A	*ARID1A*, *CTNNB1*	*ERBB2* (amp)
TCGA-BR-7196-01	PCC	10	*PIK3CA*, *ARD1A*, *BCOR*	
TCGA-BR-8366-01	PCC	5	*BRAF*, *PIK3CA*, *KRAS*, *SMAD4,* *RHOA*, *BCOR*	
TCGA-BR-8686-01	PCC	20	*PIK3CA*, *ARID1A*	
TCGA-BR-A4J4-01	PCC	5	*SPEN*	*ERBB2* (amp)
TCGA-HU-A4H0-01	PCC	5	*ERBB2*, *RHOA*	
TCGA-HU-8608-01	GCLS	80	*PIK3CA*, *ARID1A*, *JAK2, BCOR*	*JAK2* (amp), *CD274* (amp), *PDCD1LG2* (amp)
TCGA-HU-A4G2-01	GCLS	60	*PIK3CA*	*SMAD4* (del), *SMAD2* (del)

TCGA-CG-5722-01	N/A	N/A	*PIK3CA*	

amp, amplification; del, deletion
